# Programmable Hydrogels for Surgical Interface Control: Function-Based Design, DNA-Based Molecular Modules, and Translational Evaluation

**DOI:** 10.3390/gels12080695

**Published:** 2026-08-04

**Authors:** Hyun Jung Koh, Jin-Oh Jeong, Hoon Choi

**Affiliations:** 1 Department of Anesthesiology and Pain Medicine, Seoul St. Mary’s Hospital, College of Medicine, The Catholic University of Korea, 222, Banpo-daero, Seocho-gu, Seoul 06591, Republic of Korea; hjkoh92@naver.com; 2 Wake Forest Institute for Regenerative Medicine (WFIRM), Wake Forest School of Medicine, 391 Technology Way NE, Winston-Salem, NC 27101, USA; jijeong@wakehealth.edu

**Keywords:** surgical interface control, programmable hydrogels, responsive hydrogels, DNA hydrogels, wet-tissue adhesion, surgical sealants, hemostatic hydrogels, antiadhesion barriers, tissue integration, biomaterial translation

## Abstract

Surgical procedures create dynamic interfaces between tissues, fluids, gases, and applied materials. Failure to control these interfaces can cause leakage, postoperative adhesion, scar tethering, poor tissue integration, maladaptive host responses, or loss of mechanical support. Hydrogels are attractive surgical materials because their hydrated polymer networks can be engineered for wet-tissue conformity, adhesion, transport, degradation, mechanical compatibility, and local biological activity. However, many hydrogel systems are still evaluated by polymer chemistry, stimulus type, or isolated physicochemical properties rather than by the operative function required at a defined surgical boundary. This narrative review proposes a function-based framework for designing and evaluating programmable hydrogels in surgical-interface control. Four principal functions—sealing, separation, protection, and integration/reinforcement—are linked to dominant failure modes, design priorities, endpoints, and comparator requirements. Hemostatic and other biological activities are treated as primary clinical claims or adjunct programs when they support these interface functions. Responsiveness is distinguished from clinically meaningful programmability using five operational criteria: input relevance, encoded transition, baseline and off-target stability, interface-level output, and matched-control comparison. DNA-based hydrogels are discussed as molecular modules for recognition, assembly, crosslinking, degradation, actuation, and release, mainly within mechanically robust hybrid systems. This framework emphasizes time-resolved, function-specific evaluation under procedure-relevant conditions.

## 1. Introduction

Surgery creates, exposes, or modifies interfaces between tissues, body fluids, gases, and applied materials. These interfaces are not passive anatomical boundaries. They are dynamic healing environments in which mechanical loading, fluid exposure, inflammation, extracellular matrix (ECM) remodeling, cellular infiltration, and host–material interactions change over time [1,2,3]. Effective surgical interface control may require sealing of a disrupted tissue boundary, temporary separation of injured surfaces, protection of vulnerable structures, or integration and reinforcement of a tissue–material contact. When these requirements are not adequately achieved, interface failure may appear as leakage, dehiscence, postoperative adhesion, scar-related tethering or compression, poor tissue incorporation, loss of mechanical support, or maladaptive foreign-body response [2,4,5]. Complications that are usually considered separately across surgical specialties may therefore share a common design problem: failure to establish, maintain, and resolve an appropriate surgical interface.

Existing surgical materials already address many of these functions. Fibrin-based and synthetic sealants can support hemostasis, reinforce closure, or reduce leakage [5], whereas bioresorbable barriers can temporarily separate injured surfaces to reduce postoperative adhesion [6]. These materials remain clinically valuable when the operative requirement is well defined and when the desired material behavior remains relatively stable during the relevant healing interval. Hydrogels are attractive in this context because their hydrated polymer networks can be engineered across broad ranges of stiffness, viscoelasticity, porosity, permeability, swelling, degradation, and tissue adhesion [7]. Their compliant and water-rich structure can facilitate contact with wet or irregular tissue surfaces [8], and injectable, sprayable, or in situ-forming formulations may enable delivery into confined defects or minimally invasive operative fields [9]. These features do not make hydrogels inherently superior to established surgical materials; rather, they make hydrogels adaptable platforms for matching material behavior to interface-specific surgical functions.

Hydrogel versatility alone, however, does not solve the problem of surgical interface control. In many conventional systems, adhesion, stiffness, permeability, swelling, and degradation are largely determined by the initial formulation and crosslinking architecture [7,10]. Such predefined behavior may be adequate when the required function is simple, localized, and time-limited, but it may become mismatched as biochemical, inflammatory, enzymatic, fluidic, cellular, or mechanical conditions evolve during healing [1,2,3]. A sealant may require rapid early adhesion and cohesive integrity, followed by degradation that does not impede tissue motion or remodeling. A separation barrier may need local retention during the adhesion-forming period but should not persist long enough to promote encapsulation or interfere with re-entry. A reinforcing matrix may initially support load transfer but later permit cellular infiltration, vascularization, ECM deposition, and tissue remodeling [5,6,10,11]. The limitation is therefore not that static hydrogels are intrinsically inadequate, but that a fixed material state may not match the changing temporal and spatial requirements of a healing surgical interface.

Responsive hydrogels provide one route to address this mismatch by altering adhesion, permeability, mechanics, degradation, surface behavior, or molecular release after a biochemical or operator-delivered input [10,12]. For surgical applications, however, responsiveness is useful only when the input is present or controllable at the operative site, the transition occurs with appropriate timing and specificity, and the resulting output improves a defined interface-level function. In this review, programmability refers to the intentional encoding of temporal, spatial, threshold-dependent, sequential, or logic-like material transitions into a hydrogel system. A programmable surgical-interface hydrogel is therefore best evaluated by whether an encoded transition improves a function-specific outcome compared with an appropriate nonresponsive or nonprogrammed material with similar baseline properties.

Deoxyribonucleic acid (DNA)-based hydrogels provide an informative model of molecular programmability. Nucleic-acid sequences can function as structural components, programmable crosslinkers, recognition elements, degradable linkers, and signal-responsive modules, allowing sequence-level design to regulate self-assembly, gelation, network architecture, viscoelastic behavior, degradation, actuation, and cargo release [13,14,15,16,17,18]. Functional nucleic-acid motifs can further translate defined molecular inputs into changes in network composition, stiffness, integrity, or therapeutic output [15,16]. A recent implantable DNA-based hydrogel designed to detect and respond to postoperative rehemorrhage illustrates the potential relevance of such molecular programming to an operative setting [19]. At the same time, DNA-based hydrogels face translational barriers that include limited mechanical robustness, susceptibility to nuclease degradation, possible immunological activity, cost and scalability, sterilization, storage, and intraoperative handling [17,18]. For this reason, DNA-based hydrogels are better viewed not as immediate replacements for conventional surgical sealants, barriers, or reinforcement materials, but as programmable molecular modules that may provide recognition, sensing, release, degradation, or remodeling functions within mechanically stronger hydrogel or composite systems.

Prior reviews have provided complementary perspectives on programmable hydrogels, tissue adhesives, antiadhesion materials, and DNA-based hydrogel systems [4,5,6,10,11,12,14,17,18]. However, no single framework has integrated these domains by beginning with cross-specialty surgical-interface failure modes and then deriving the required material functions, programmed behaviors, and evaluation strategies. The relationship between the present framework and these earlier review traditions is examined comparatively in Section 1.2.

This narrative review develops a function-based framework for programmable hydrogels in surgical-interface control. Rather than organizing the field by polymer chemistry, stimulus type, molecular mechanism, or anatomical specialty, it begins with operative failure modes and four principal interface functions: sealing, separation, protection, and integration/reinforcement. The review then links hydrogel design requirements, programmability criteria, representative surgical applications, DNA-based molecular modules, and translational evidence requirements to these functions. The goal is to clarify how programmability claims should be connected to defined surgical-interface functions and procedure-relevant evidence, not to rank hydrogel chemistries or provide an exhaustive catalogue of responsive materials.

### 1.1. What This Review Adds

This review contributes three interface-directed perspectives to the programmable hydrogel literature.

- It organizes surgical hydrogels according to required interface function and failure mode rather than polymer chemistry, stimulus type, or anatomical specialty.
- It defines clinically meaningful programmability using operational criteria that link input relevance, encoded material transition, baseline and off-target stability, interface-level output, and appropriate comparator selection.
- It positions DNA-based hydrogels as molecular programming modules and connects programmable hydrogel claims to translational evidence requirements, including operative handling, sterilization, storage, safety, and procedure-specific benchmarking.

### 1.2. Comparative Positioning Against Existing Reviews

Existing reviews provide essential but largely complementary maps of hydrogel science. Broad hydrogel reviews commonly organize the literature according to polymer source or backbone, network and crosslinking architecture, delivery format, physicochemical property, or biomedical application [7,8,9]. Reviews specifically addressing programmable or stimuli-responsive hydrogels have instead emphasized the origin of the input, the resulting material transition, and broad application domains. For example, programmable-hydrogel frameworks have classified biological, chemical, and physical stimulation and subsequently discussed changes in dimensions, mechanical support, cell attachment, and molecular sequestration, whereas stimuli-responsive-hydrogel perspectives have emphasized dynamic matrices, shape morphing, and four-dimensional bioprinting for tissue engineering [10,12]. These approaches explain how hydrogels are constructed or how they change, but the operative complication to be prevented and the function-specific evidence required to justify that change are not their primary organizing axes.

More clinically focused reviews have addressed important components of the surgical-interface problem. Reviews of tissue adhesives have examined wet adhesion, multiscale interfacial mechanisms, spatiotemporal selectivity, tissue-specific environments, and barriers to clinical translation [5,11]. However, an adhesion-centered framework does not encompass operative situations in which low or asymmetric adhesion, lubricity, temporary separation, or remodeling-permissive integration is the desired outcome. Reviews of postoperative adhesion prevention have connected the biological mechanisms of adhesion formation with barrier and drug-delivery strategies [4,6], but their scope is necessarily centered on a single complication and therefore does not unify adhesion formation with leakage, washout, scar-related tethering, delamination, poor tissue ingrowth, or loss of mechanical support.

DNA-hydrogel reviews provide a different and molecularly detailed perspective by organizing the field around sequence-defined network architecture, polymer dynamics, functional nucleic-acid motifs, stimulus categories, biosensing, actuation, and controlled release [14,17,18]. These analyses establish the molecular programmability of DNA-containing systems, but they do not generally begin by asking which operative boundary is failing, which interface function is required, or whether a sequence-encoded transition improves a procedure-specific endpoint under blood exposure, lavage, pressure, motion, or other surgical constraints. Thus, across otherwise complementary review traditions, the recurring gap is not a lack of material sophistication, but the absence of a common framework that makes the pathway from surgical failure mechanism to hydrogel design and evaluation explicit.

The present review addresses this gap through a reverse-design framework. The analysis begins with the physical boundary and dominant failure mode, assigns one or more of four core interface functions—sealing, separation, protection, and integration/reinforcement—and then derives the required interfacial, mechanical, transport, degradation, retention, and handling characteristics. Only after the required function has been defined are responsive or DNA-based molecular modules evaluated for their ability to encode a clinically relevant input–transition–output relationship. The final step is the selection of function-specific endpoints, matched nonresponsive or nonprogrammed controls, and procedure-relevant translational models. This ordering also exposes design trade-offs that chemistry- or stimulus-first taxonomies may obscure: strong adhesion may benefit sealing but compromise separation, whereas prolonged persistence may support reinforcement but hinder later remodeling or surgical re-entry. Table 1 summarizes the comparative positioning of the present framework.

### 1.3. Scope and Literature Selection

This narrative review was designed to develop a function-based framework for evaluating programmable hydrogels as surgical-interface materials. It is framework-oriented rather than systematic and does not attempt quantitative synthesis, formal risk-of-bias assessment, or exhaustive ranking of hydrogel chemistries. The literature search was used to identify representative studies, mechanisms, and translational issues relevant to surgical-interface control.

Relevant publications were identified through targeted searches of PubMed, Web of Science, Scopus, and Google Scholar. The literature search was last updated in July 2026. Search terms were used in combinations and included “hydrogel,” “responsive hydrogel,” “programmable hydrogel,” “smart hydrogel,” “DNA hydrogel,” “nucleic acid hydrogel,” “tissue adhesive,” “wet-tissue adhesion,” “surgical sealant,” “hemostatic hydrogel,” “antiadhesion barrier,” “wound healing,” “tissue integration,” “biomaterial translation,” “sterilization,” and “regulatory evaluation.” Additional references were identified from the bibliographies of relevant articles when they contributed to the conceptual framework or provided representative examples of interface-specific hydrogel performance.

Studies were discussed when they contributed to one or more of three aims: defining surgical-interface functions and failure modes; illustrating hydrogel mechanisms that could support temporal, spatial, threshold-dependent, sequential, or logic-like material behavior; and identifying translational evidence requirements for surgical use. Representative studies were prioritized when they included direct evaluation of a surgical or tissue-mimetic interface, testing under wet or mechanically relevant conditions, comparison with a nonresponsive or nonprogrammed control, or assessment of operative usability, degradation, safety, sterilization, storage, or other translational constraints.

Priority was given to peer-reviewed original studies that included material characterization together with ex vivo, in vivo, or procedure-relevant functional endpoints. High-quality reviews, perspective articles, and regulatory or standards-related documents were included when they clarified terminology, summarized established material classes, or informed translational evaluation. Studies focused only on nonsurgical drug delivery, cell culture, or generic stimulus responsiveness were not emphasized unless they provided a mechanism or design principle directly relevant to surgical-interface control. Accordingly, the selected examples should be interpreted as illustrative rather than exhaustive.

## 2. Surgical Interfaces as Function-Defined Boundaries

Surgical interfaces can be understood through two complementary dimensions: the physical boundary involved and the function required at that boundary. The physical boundary may occur between adjacent tissues, between tissue and a gas- or fluid-filled space, or between tissue and an applied material. In this review, the required interface function is organized around four principal mechanical or structural objectives: sealing, separation, protection, and integration/reinforcement. Biological activities such as hemostasis, antimicrobial action, immunomodulation, angiogenic support, or local disease surveillance are not excluded from this framework; rather, they are interpreted according to the interface function they support. Hemostasis may represent the primary clinical indication of a surgical hydrogel, but it should still be mapped to the interface problem it helps solve, such as sealing a disrupted tissue–fluid boundary, protecting a vulnerable bleeding surface, or supporting reinforcement through clot formation and material fixation. These dimensions are distinct but clinically interdependent. A single operative field may contain several boundary types, and the dominant interface function may change as healing progresses. Defining surgical interfaces in this way shifts material evaluation away from isolated physicochemical properties and toward operative failure modes.

### 2.1. Physical Boundaries and Interface Functions

The first dimension concerns where the material must act. Tissue–tissue interfaces arise where biological surfaces are approximated, separated, or exposed to one another during healing, as occurs along peritoneal, pericardial, tendon, and nerve-associated surfaces [4,20,21,22]. Tissue–gas/fluid interfaces occur where disrupted tissues must contain air, cerebrospinal fluid, blood, exudate, or luminal contents, as illustrated by pulmonary air leaks, dural defects, vascular or hemostatic surfaces, and gastrointestinal anastomoses [23,24,25]. Tissue–material interfaces are created whenever native tissue contacts an applied biomaterial, including sealants, barriers, patches, meshes, grafts, prostheses, or hydrogels [26]. These categories describe the anatomical and physical setting of the interface, but they do not by themselves define what the material must accomplish.

The second dimension concerns the operative function required for successful interface control. Sealing limits the passage of gas, biological fluids, or luminal contents across a disrupted boundary [27]. Separation maintains temporary distance between injured surfaces to reduce postoperative adhesion or fibrotic bridging [6]. Protection preserves the mobility, gliding, structural integrity, or neurological function of vulnerable tissues, particularly around tendons and nerves, by reducing friction, compression, and scar-related tethering [21,22]. Integration/reinforcement requires stable coupling between native tissue and an applied material to provide mechanical support, biological incorporation, or both, while limiting maladaptive inflammation, delamination, and fibrotic encapsulation [26,28].

These four functions describe the dominant mechanical or structural role of a material at the surgical boundary. They do not exclude biological activity. Antimicrobial, anti-inflammatory, antioxidative, angiogenic, immunomodulatory, or local disease-control effects may be essential in selected operative contexts, but they should be interpreted as adjunct programs when they improve a defined interface function. For example, antimicrobial release may support sealing in a contaminated luminal defect, anti-inflammatory activity may support separation by reducing adhesion formation, and angiogenic or immunomodulatory cues may support integration by improving tissue incorporation.

**Figure 1:**
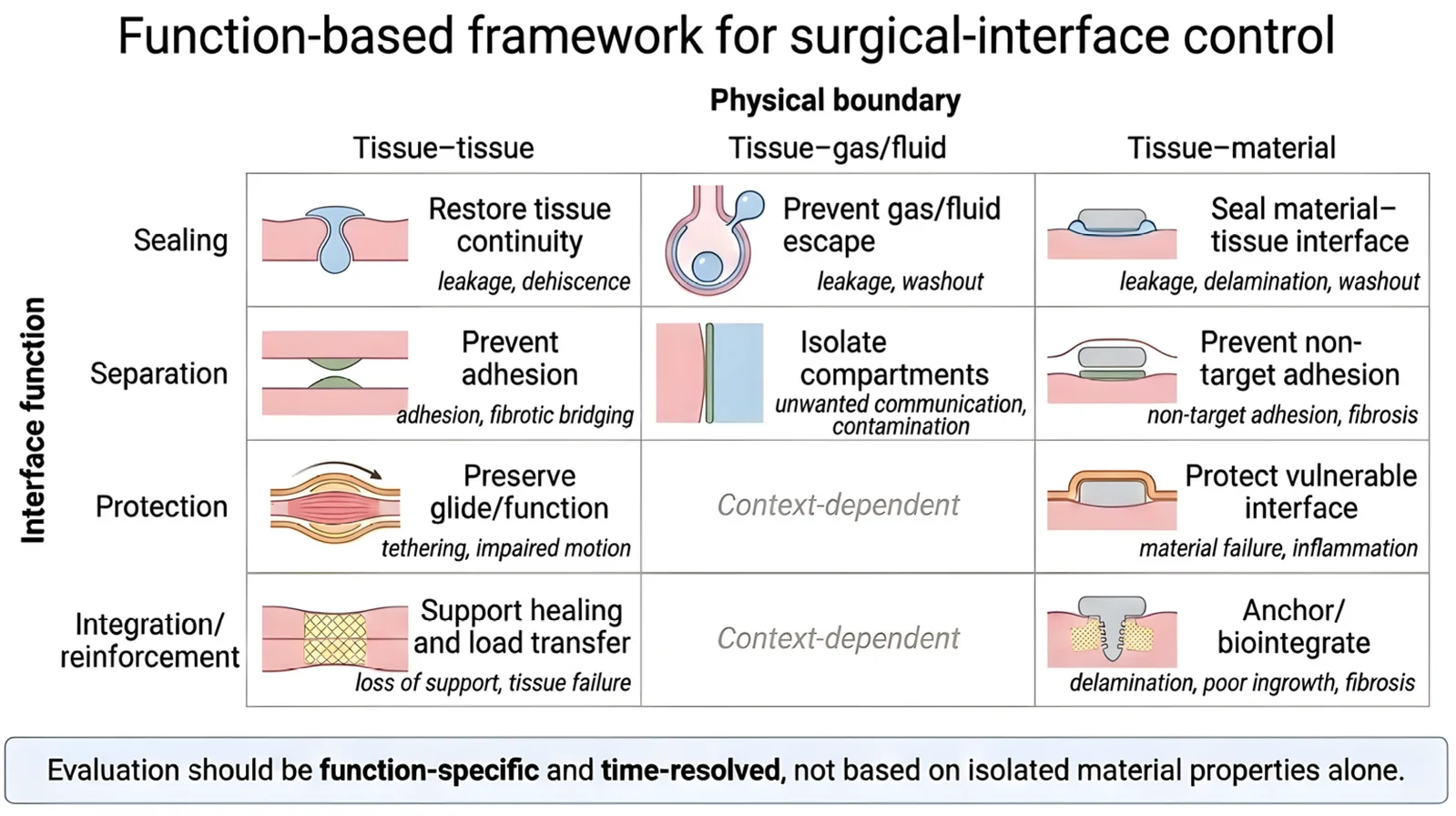
Function-based framework for surgical-interface control. Surgical interfaces are organized by physical boundary type and required interface function. Physical boundaries include tissue–tissue, tissue–gas/fluid, and tissue–material interfaces, whereas principal interface functions include sealing, separation, protection, and integration/reinforcement. Representative failure modes include leakage, washout, adhesion, fibrotic bridging, tethering, delamination, poor tissue ingrowth, fibrosis, and loss of support. Some boundary–function combinations are context-dependent. The framework emphasizes function-specific, time-resolved evaluation rather than assessment based on isolated material properties alone. Hemostatic activity is not excluded from the framework; it should be mapped to the dominant interface function it supports, such as sealing, protection, or reinforcement.

These dimensions intersect in clinically relevant ways. A thoracic operative site may include a tissue–gas/fluid boundary at the lung surface and a tissue–material boundary where a sealant or reinforcing patch is applied, with sealing as the dominant function [23]. An abdominal procedure may combine a tissue–fluid boundary at an anastomosis with tissue–tissue surfaces at risk of adhesion, requiring both containment and temporary separation [6,25]. Around a repaired tendon or peripheral nerve, the relevant boundary may be tissue–tissue or tissue–material, whereas the dominant objective is preservation of gliding, mobility, or conduction rather than pressure-driven containment [21,22]. Accordingly, evaluation of a surgical-interface hydrogel should specify both the physical boundary and the required function. Figure 1 summarizes this framework by mapping physical boundary types to interface functions and representative failure modes.

### 2.2. Interface Failure Modes and Function-Specific Priorities

Each interface function is associated with characteristic failure modes and design priorities. Sealing is required when a disrupted boundary must prevent the escape of gas, cerebrospinal fluid, blood, exudate, or luminal contents [29]. Failure may appear as persistent or recurrent leakage, dehiscence, washout, delamination, cohesive rupture, excessive swelling, or premature loss of material integrity [23,24,25]. Although pulmonary air leak, dural leakage, and gastrointestinal anastomotic leakage occur in different anatomical settings, they share several interface-level priorities: rapid deployment, conformal contact with wet tissue, sufficient interfacial adhesion, cohesive durability under pressure or motion, controlled swelling, and degradation that does not outpace native repair [29,30,31].

Separation is required when injured surfaces must remain apart during the early phase of healing. Unlike sealing, separation does not primarily depend on strong attachment across a defect. Instead, a barrier must cover vulnerable surfaces, remain at the intended site, reduce protein fouling and cellular attachment, and limit tissue bridging during the adhesion-forming period [32,33]. Failure may permit fibrotic connections that restrict tissue mobility, distort anatomy, or complicate re-entry [33]. Separation therefore depends not only on antiadhesive chemistry, but also on deployment accuracy, local retention, surface selectivity, swelling control, and appropriately timed degradation [20,32,34].

Protection is required when a repaired or exposed structure must preserve mobility, gliding, structural integrity, or neurological function during healing. Although protection may include physical separation, its endpoint is functional preservation rather than prevention of tissue contact alone. Around tendons and peripheral nerves, a protective interface must provide conformal and nonconstrictive coverage, reduce friction and scar-related tethering, accommodate repetitive motion, and remain stable without interfering with intrinsic repair [35,36]. Failure may manifest as restricted tendon excursion, impaired nerve gliding, pain, neurological dysfunction, or motor impairment [35,36,37,38].

Integration/reinforcement is required when an applied material must remain coupled to native tissue while providing mechanical support, biological incorporation, or both. Immediate attachment is not sufficient for this function. Early fixation must evolve into durable load transfer, tissue ingrowth when intended, controlled remodeling, and an acceptable host response [26,39]. Failure may appear as displacement, delamination, seroma, shrinkage, poor tissue incorporation, chronic inflammation, fibrotic encapsulation, or loss of structural support [39,40,41]. Integration/reinforcement therefore requires coordinated control of fixation kinetics, mechanical compatibility, fatigue resistance, material persistence, host remodeling, and tissue ingrowth.

### 2.3. Functional Trade-Offs as the Rationale for Programmable Interfaces

Surgical interfaces rarely require a single isolated function. A material may need to adhere securely to a target surface while remaining nonadhesive toward adjacent tissues, persist long enough to provide support yet degrade before it interferes with remodeling, and withstand physiological loading while remaining mechanically compatible with moving tissue. These trade-offs are evident in pericardial barriers, which must remain on the cardiac surface without promoting adhesion to surrounding structures [20], and in tendon or nerve wraps, which must retain position while preserving gliding and avoiding constriction [35,36]. Sealing systems face related balances among interfacial adhesion, cohesive durability, swelling, compliance, and degradation kinetics [29,31,42]. Surgical-interface performance therefore cannot be inferred from a single metric such as adhesion strength, elastic modulus, swelling ratio, or degradation time.

These competing requirements also vary across regions of the same interface and across successive phases of healing. A barrier may require selective retention at the target surface while minimizing interaction with opposing tissues. A sealant may require rapid initial attachment followed by reduced material persistence as native repair progresses [11,20,31]. A reinforcing matrix may initially contribute to load sharing but later need to permit tissue infiltration and remodeling without provoking excessive fibrosis [39,40,41,43]. Uniform material behavior and single-time-point testing may therefore obscure clinically relevant differences in performance.

Dynamic and programmable hydrogels offer a strategy for addressing these spatial and temporal trade-offs. Conventional hydrogels can be tuned to satisfy one or more interface requirements, but predefined adhesion, crosslinking, swelling, mechanics, and degradation may impose compromises when different regions or healing phases require different behaviors [43,44]. By contrast, programmable systems can be designed so that selected outputs, such as adhesion, permeability, stiffness, degradation, molecular release, or surface selectivity, change according to encoded timing, spatial organization, thresholds, sequential logic, or local biological signals [10,11,43,44,45]. Their value is determined by performance in the intended biological and mechanical environment, not by dynamic behavior itself. This functional logic provides the basis for evaluating hydrogel architecture, responsive mechanisms, and DNA-based molecular modules in the following sections.

## 3. Hydrogel Design Requirements for Surgical Interface Control

Hydrogel design variables are considered here only insofar as they determine performance at a surgical boundary. The relevant question is whether the final construct can be delivered through the intended access route, conform to wet tissue, resist dilution or lavage, maintain adhesion and cohesion under pressure or motion, avoid hazardous swelling, and persist for the required healing interval. Network architecture, mechanics, transport, and degradation are therefore treated as function-dependent design variables rather than as independent physicochemical classifications [7,8,29,46,47,48].

### 3.1. Network Architecture, Conformity, and Retention

Network architecture should be selected according to deployment and retention requirements. Injectable, sprayable, shear-thinning, or in situ-forming systems must remain deliverable long enough to cover the target yet stabilize rapidly enough to avoid runoff, dilution, or washout [9,32,46,48,49,50,51]. Dynamic, hybrid, or reinforced networks are relevant only when they provide an operative advantage—such as recovery after injection, energy dissipation under motion, or controlled remodeling—without compromising reproducibility, sterilization, or surgical handling [47,48,49].

After placement, retention is function specific. A sealant must resist pressure-driven separation and lavage; a separation barrier must remain target-facing without adhering to opposing tissues; a protective wrap must avoid migration and constriction during motion; and a reinforcing matrix must remain coupled to tissue while load transfer and biological incorporation develop. These requirements should be tested under blood exposure, irrigation, organ motion, and the intended duration of material action rather than inferred from crosslink chemistry alone [29,32,47,48,49,51].

### 3.2. Adhesion, Cohesion, and Mechanical Compatibility

At a surgical interface, adhesion and cohesion should be evaluated as coupled failure modes. Blood, mucus, and exudate can prevent intimate tissue contact, while pressure, cyclic deformation, or organ motion can produce interfacial delamination or cohesive rupture after attachment [29,51,52,53,54]. Accordingly, evaluation should prioritize wet-field attachment, washout resistance, cyclic or pressure loading, fatigue behavior, and compatibility with tissue deformation rather than cataloguing adhesive functional groups or reporting a single peak-strength value.

The required balance remains function-dependent. Sealing requires rapid attachment and cohesive durability; separation requires selective retention with low non-target adhesion; protection requires lubricity, compliance, and preserved gliding; and integration/reinforcement requires fixation, fatigue resistance, and load transfer compatible with tissue remodeling. Test selection should therefore follow the intended failure mode and interface function [3,29,42,51,52].

### 3.3. Swelling, Degradation, and Material Lifetime

Swelling, transport, and degradation are retained here as site-specific safety and material-lifetime variables. Excessive fluid uptake can weaken attachment, distort the treated field, or compress vulnerable structures in confined spaces, whereas premature degradation can cause recurrent leakage, barrier failure, or loss of support [31,55,56]. Conversely, prolonged persistence may obstruct tissue motion, remodeling, or surgical re-entry and may sustain an undesirable foreign-body response [31,39,55,57].

Acceptable behavior depends on the operative function and healing interval. A sealant should preserve containment during the period of leak vulnerability; a separation barrier should remain through the adhesion-forming window and then clear; a protective interface should preserve motion without late constriction; and a reinforcing matrix should maintain load transfer until tissue incorporation is sufficient. Evaluation should therefore measure volumetric change, residual mechanical or barrier function, and degradation over time in relevant biological fluids rather than report equilibrium swelling or mass loss in isolation.

### 3.4. Operative Handling and Deliverability

Operative handling links material design to clinical usability. A surgical-interface hydrogel must be prepared reproducibly, delivered accurately, and converted into a stable material within the constraints of the operative field. Precursor viscosity, shear-thinning behavior, sprayability, working time, gelation kinetics, curing mechanism, and device compatibility determine whether a formulation can reach a confined defect, spread across an irregular surface, or be applied through minimally invasive instruments [9,49,50,58]. A formulation that performs well in vitro may fail clinically if it requires prolonged preparation, has a narrow working window, obstructs the operative view, cannot be delivered through available applicators, or is displaced before gelation.

Working time and stabilization kinetics require particular attention. Rapid gelation may limit spreading, repositioning, or passage through a delivery device, whereas delayed gelation may permit dilution, runoff, washout, or migration before fixation occurs. Systems requiring component mixing, specialized applicators, external curing, light exposure, ultrasound, or other triggers introduce additional variables, including mixing ratio, applicator clogging, curing depth, target access, trigger dose, tissue heating, and operator-to-operator variability [9,50,58]. These issues are not secondary technical details; they determine whether a programmable material can actually be used at the intended surgical boundary.

Practical evaluation should include preparation time, delivery accuracy, working and curing times, retention before and after gelation, compatibility with open and minimally invasive instruments, visibility of the treated field, performance in the presence of blood or lavage fluid, and the ability to correct or remove material after misapplication [46,58]. For programmable hydrogels, handling assessment should also test whether the programmed input can be delivered reliably in the operative context and whether the transition remains spatially confined to the intended interface.

### 3.5. Linking Material Properties to Surgical-Interface Functions

The suitability of a hydrogel for surgical-interface control cannot be determined from any single material property. Each function requires a distinct combination of interfacial, mechanical, transport, degradation, retention, and handling characteristics. Sealing prioritizes wet-tissue contact, adhesion, cohesive durability, pressure resistance, controlled swelling, and repair-matched degradation. Separation prioritizes conformal coverage, selective retention, low adhesion to opposing tissues, limited fouling or cellular attachment, dimensional stability, and controlled persistence. Protection prioritizes nonconstrictive coverage, lubricity, mechanical compliance, tolerance of repetitive motion, and preservation of gliding or conduction. Integration/reinforcement prioritizes fixation, load transfer, fatigue resistance, tissue ingrowth when intended, controlled remodeling, and favorable host response.

These profiles overlap but are not interchangeable. Optimizing one property may improve one function while compromising another. Candidate materials should therefore be evaluated against a predefined interface objective and a corresponding set of material, biological, and operative performance criteria. Table 2 summarizes function-specific design priorities, principal failure modes, and minimum evaluation endpoints for surgical-interface hydrogels.

## 4. From Responsiveness to Programmability in Surgical Interfaces

Responsive hydrogels can change adhesion, permeability, mechanics, degradation, surface behavior, or release after a biochemical or operator-controlled input [59]. In surgical applications, however, responsiveness is relevant only when the input is present or deliverable at the operative site and the resulting transition improves a defined interface function [45,60,61]. Programmability is therefore treated as an encoded and testable design relationship rather than as a catalogue of stimulus classes.

### 4.1. Distinguishing Responsiveness, Adaptiveness, and Programmability

Responsiveness, adaptiveness, and programmability describe related but distinct levels of material control. Responsiveness refers to a material-state change triggered by a defined input. In hydrogels, this may involve changes in swelling, network density, stiffness, permeability, degradation, adhesion, surface behavior, or molecular release after exposure to chemical or physical stimuli [59]. Adaptiveness implies that the response improves material–environment matching over time, for example, by allowing mechanics, transport, adhesion, or degradation to shift as the local microenvironment evolves [44,60]. Programmability adds a further design requirement: the timing, threshold, sequence, spatial distribution, or combination of outputs must be intentionally encoded into the material system [10,11,45].

A material that changes state after a generic cue is responsive, but it is not necessarily programmable. For surgical use, the term should be reserved for systems in which a clinically relevant input is intentionally linked to a timed, localized, threshold-dependent, sequential, or logic-like transition that improves an interface-level outcome [10,45,59,62]. Table 3 summarizes the operational criteria used in this review to evaluate such claims.

Several illustrative examples clarify how these criteria distinguish generic responsiveness from clinically meaningful programmability. First, a pH-responsive hydrogel that swells or releases a payload in an acidic buffer is responsive, but it should not be considered programmable for surgical use unless the relevant pH range is present or controllably generated at the target interface, the transition occurs at an intentionally designed threshold and time scale, off-target activation is limited, and the resulting change improves a function-specific endpoint compared with a trigger-insensitive control. Second, an enzyme-degradable hydrogel that exhibits accelerated mass loss in a protease solution remains merely responsive when evaluation is limited to degradation kinetics. The same mechanism supports a programmability claim when clinically relevant enzyme levels activate an encoded cleavage threshold during the intended healing interval, baseline and non-target stability are demonstrated, and degradation or payload release improves sealing, separation, protection, or integration relative to a non-cleavable analogue [45,59]. Third, Janus hydrogels with a target-facing adhesive surface and a non-target-facing antiadhesive or antifouling surface illustrate spatial programmability when the directional architecture is intentionally encoded, maintained after deployment, and shown to preserve fixation while reducing tissue bridging compared with uniform or reversed-orientation controls [63,64,65,66]. An implantable DNA hydrogel designed to detect and respond to postoperative rehemorrhage provides a molecularly explicit example of event-linked programming, because a clinically relevant hemorrhage-associated input is coupled to designed diagnostic and therapeutic outputs [19]. Under the present framework, however, even such an advanced system requires evidence of input specificity, response threshold, off-target stability, interface-level benefit, and comparison with recognition-deficient or nonresponsive controls before a clinically meaningful programmability claim is fully supported. Thus, responsiveness and programmability are not fixed labels determined by hydrogel chemistry alone; they describe different levels of evidence connecting an input, an encoded material transition, and a surgical-interface outcome.

### 4.2. Operative Inputs and Input–Transition–Output Logic

Designing a programmable surgical-interface hydrogel should begin with an operative event or failure risk rather than with a generic stimulus category. Inputs of direct surgical relevance include hemorrhage- or coagulation-associated signals, inflammatory mediators, protease or nuclease activity, microbial or tissue-injury signals, and operator-controlled triggers that can be delivered within the operative field. Blood, exudate, tissue debris, cautery injury, irrigation or lavage, background enzymes, and organ motion must be treated as competing conditions that may dilute, mask, or falsely activate the programmed response [19,45,59,67,68,69,70].

An input is clinically meaningful only when its concentration, timing, and spatial distribution at the target boundary are defined and when the resulting transition improves sealing, separation, protection, or integration/reinforcement. Enzyme- or nuclease-sensitive modules may control degradation or molecular release, whereas hemorrhage-responsive recognition may couple rebleeding to local stabilization, diagnostic signaling, or therapeutic delivery [19,67,68,69,70]. These mechanisms should be tested with non-cleavable, recognition-deficient, trigger-insensitive, or nonprogrammed controls under blood exposure, lavage, and relevant off-target biochemical conditions.

**Figure 2:**
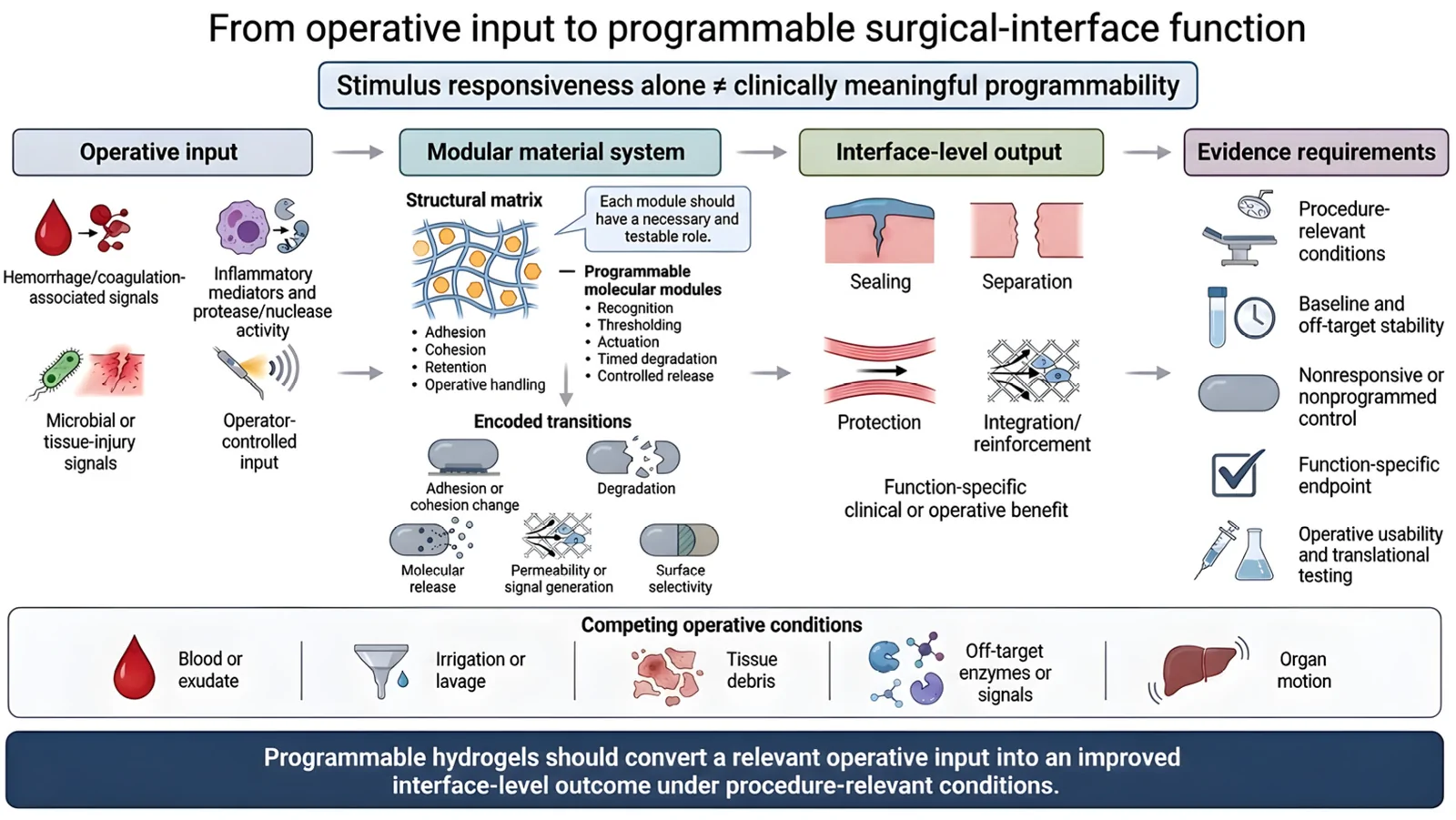
From operative input to programmable surgical-interface function. Clinically meaningful programmability requires more than generic stimulus responsiveness. Relevant operative inputs include hemorrhage/coagulation-associated signals, inflammatory mediators and protease/nuclease activity, microbial or tissue-injury signals, and operator-controlled triggers. These inputs act on a modular material system in which a structural matrix provides adhesion, cohesion, retention, and operative handling, whereas programmable molecular modules provide recognition, thresholding, actuation, timed degradation, or controlled release. The resulting encoded transitions should improve one or more interface-level functions—sealing, separation, protection, or integration/reinforcement—under competing operative conditions such as blood or exudate, irrigation or lavage, tissue debris, off-target enzymes or signals, and organ motion. Supporting evidence should demonstrate performance under procedure-relevant conditions, baseline and off-target stability, comparison with appropriate nonresponsive or nonprogrammed controls, improvement in function-specific endpoints, and operative usability and translational testing.

Operator-controlled inputs such as light or ultrasound should be retained only when they can be delivered with adequate access, localization, and safety. This operative filtering favors modular architectures: a mechanically robust hydrogel or composite matrix provides adhesion, cohesion, retention, and handling, whereas DNA, aptamer, DNAzyme, clustered regularly interspaced short palindromic repeats (CRISPR)-responsive, or enzyme-sensitive modules provide recognition, thresholding, actuation, degradation, or release. Each module should have a necessary and separately testable role, and the complete system should advance through the staged translational evaluation pathway described in Section 7. Figure 2 summarizes this input–transition–output logic and the evidence needed to distinguish material responsiveness from programmable interface function.

### 4.3. Electroactive, Piezoelectric, Ionic–Electronic, and Mechanoresponsive Programming

Electrical and mechanical inputs provide a distinct route to programmable surgical-interface control that complements biochemical recognition and operator-delivered optical or acoustic triggers. Electroactive hydrogels broadly refer to hydrated networks that support, modulate, or respond to electrical charge transport or applied electrical fields. Within this group, electronically conductive or semiconducting components can facilitate electronic charge transport [71,72], whereas ionically conductive hydrogels transmit charge primarily through mobile ions in the water-rich network [73]. Ionic–electronic hydrogel interfaces are designed to couple ion-dominated biological tissues with electronic recording or stimulation devices while maintaining soft mechanical contact, low interfacial impedance, and stable electrical coupling [73,74]. Piezoelectric systems convert mechanical deformation into electrical polarization and may therefore use physiological motion, applied pressure, or ultrasound as an activating input [75,76]. Mechanoresponsive hydrogels encompass a broader category in which strain, compression, shear, or cyclic loading alters enzyme activity, network structure, stiffness, degradation, molecular exposure, or cargo release [77,78]. These categories overlap but are not interchangeable, and their relevance to surgery depends on whether the electrical or mechanical transition improves a defined interface function under the expected operative and physiological loading conditions.

Electroactive and ionic–electronic hydrogels are particularly relevant when a surgical interface must record endogenous physiological activity, deliver electrical stimulation, or maintain communication between ionically conducting tissue and electronic hardware. In these systems, the hydrogel may serve as a compliant ionic conductor, incorporate an electronically conducting or semiconducting phase, or combine both modes of charge transport to reduce mechanical and electrical discontinuity at the tissue–device boundary [71,72,73]. Recent conducting-polymer hydrogels have supported conformal all-hydrogel interfaces for long-term electrophysiological recording and stimulation, whereas semiconducting hydrogels have enabled active sensing and amplification of physiological signals [71,72]. Injectable conductive bioadhesive hydrogels further demonstrate how electrical function can be combined with wet-tissue adhesion, anti-swelling stability, and atraumatic fixation to establish low-impedance coupling with fine peripheral nerves [74]. Miniaturized multifunctional hydrogel probes integrating optical and electrical components illustrate an additional route toward simultaneous stimulation and recording [79]. For surgical-interface control, however, conductivity alone is insufficient. Evaluation should include deployability, tissue and device adhesion, dimensional stability after hydration, frequency-dependent impedance, electrochemical durability, recording fidelity, stimulation efficiency, stability under cyclic deformation, and chronic tissue response.

Piezoelectric and mechanoresponsive systems use mechanical energy as either the activating input or the regulated output. Piezoelectric hydrogel composites can convert ultrasound, tissue deformation, pressure, or cyclic motion into localized electrical stimulation without permanently implanted power leads. In peripheral-nerve repair, ultrasound-responsive piezoelectric nanofiber–hydrogel conduits have combined wireless electrical stimulation with controlled growth-factor release, whereas an injectable piezoelectric antiadhesive hydrogel has coupled ultrasound-induced stimulation with physical separation of the repaired tendon from surrounding tissues [75,76]. Mechanoresponsive programming is broader and does not necessarily involve electrical conversion. Mechanical deformation may expose or separate molecular components, alter enzyme activity, reorganize the network, or regulate cargo transport. For example, strain-mediated disruption of an engineered thrombin–hirudin interaction has been used to reversibly activate an embedded enzyme program and produce hydrogel self-stiffening or self-softening [77]. Tissue-adherent hybrid hydrogel actuators have also enabled localized drug release that varies with actuation magnitude, frequency, and duration while maintaining conformal attachment during repeated deformation [78]. These systems are most relevant to surgical interfaces when the triggering load reflects physiological motion or a controllable operative input and when the resulting electrical, mechanical, or therapeutic output improves sealing, separation, protection, or integration/reinforcement.

Electrical conductivity, piezoelectricity, or sensitivity to deformation does not by itself establish clinically meaningful programmability. The relevant input should be present at the intended interface or delivered through a controllable workflow, and the relationship among input magnitude, response threshold, transition kinetics, spatial localization, and functional output should be characterized under physiologically relevant loading conditions. Electroactive systems should be tested for frequency-dependent impedance, charge-injection or stimulation performance, signal fidelity, electrochemical stability, and resistance to hydration- or motion-induced drift. Piezoelectric and mechanoresponsive systems additionally require calibration across strain, pressure, loading frequency, and cycle number, together with assessment of response reversibility, fatigue stability, unintended activation, and output persistence after the mechanical input is removed [71,72,73,74,75,76,77,78,79]. Appropriate controls may include mechanically matched nonconductive systems, nonpiezoelectric composites, inactive molecular modules, and sham electrical, ultrasound, or mechanical stimulation. Most importantly, the programmed transition should improve a function-specific interface endpoint, such as stable recording, lower stimulation threshold, preserved tissue motion, controlled release, reduced adhesion, accelerated functional recovery, or durable tissue integration, rather than merely producing a measurable electrical or mechanical signal.

### 4.4. Temporal Programming of Interface Functions

Temporal programming addresses the fact that the functional requirements of a surgical interface change during healing. Immediately after surgery, a material may need to provide rapid adhesion, sealing, hemostatic support, antimicrobial protection, or mechanical stabilization [30]. During the inflammatory and reparative phases, the same material may need to maintain temporary separation, regulate molecular transport, modulate local inflammation, or preserve tissue motion [28,33,35,36]. At later stages, excessive persistence, stiffness, adhesion, or impermeability may become detrimental if it interferes with tissue infiltration, remodeling, re-entry, or resolution of the interface [39,44]. Temporal programmability is therefore best understood as the design of a functional trajectory rather than simply delayed release or time-dependent degradation [60,61].

Early temporal programs should prioritize interface stabilization. For sealing, this may require fast wet-tissue adhesion, cohesive integrity, and resistance to washout before intrinsic tissue repair has developed [29,51]. For separation or protection, early performance may instead require conformal coverage, local retention, reduced friction, or suppression of cellular attachment during the adhesion-forming period [33,35]. When contamination, ischemic injury, or marked inflammation is relevant to the operative bed, an early antimicrobial, antioxidative, or inflammation-modulating program may be justified; however, such adjunct outputs should be included only when they support the defined interface function and are evaluated in a procedure-relevant model [80,81,82,83].

Later temporal programs should support interface resolution or remodeling rather than continued material dominance. For sealing interfaces, this may mean maintaining containment until native tissue strength is restored, followed by degradation that avoids chronic foreign-body response or mechanical restriction [31,39]. For separation and protection interfaces, late-stage success may require loss of barrier persistence, reduced adhesiveness, increased lubricity, or restoration of tissue-plane mobility after the adhesion-forming period has passed [35,36]. For integration/reinforcement, by contrast, the desired transition may be more gradual: the hydrogel may initially support load transfer and later become permissive to cellular infiltration, ECM deposition, vascularization, and tissue remodeling [28,84]. Spatiotemporally programmed macroporous hydrogels for bone regeneration [84], biodegradable microporous polyethylene glycol hydrogels that permit bone-cell network formation and matrix remodeling [85], and mechanically regulated hydrogel degradation [86] illustrate how material lifetime, degradation, and remodeling can be linked to tissue formation or local loading. The value of a temporal program lies in whether it preserves the required early function while allowing appropriate later resolution or incorporation.

### 4.5. Spatial and Asymmetric Programming

Spatial programming addresses the fact that different regions of the same surgical interface may require different material behaviors [11]. A hydrogel placed at an operative site may need to adhere strongly to a target tissue while minimizing adhesion, fouling, or friction toward adjacent structures, as illustrated by antiadhesion barriers, pericardial barriers, and motion-preserving tendon or nerve interfaces [20,33,35,36]. A reinforcing material may need localized fixation or mechanical support at a defect-facing region while preserving compliance, permeability, or tissue mobility elsewhere [39,42,84]. This requirement is particularly important when sealing and separation coexist, because indiscriminate adhesion can stabilize a target defect but simultaneously promote unwanted tissue bridging [11,33]. Spatial programmability therefore extends beyond geometric conformity: it requires intentional localization of adhesion, antiadhesion, mechanics, degradation, or molecular release within defined regions of the material.

Janus and asymmetric hydrogel systems provide a direct strategy for encoding spatially differentiated functions. In these designs, one surface can be engineered for wet-tissue adhesion or defect fixation, whereas the opposite surface can resist protein adsorption, cellular attachment, tissue bridging, or friction [63,64]. This architecture is relevant to gastrointestinal and peritoneal interfaces, where a material may need to adhere to an injured bowel surface while avoiding adhesion to adjacent abdominal tissues [63]. Janus adhesive hydrogel sheets, microstructured Janus bioadhesives, charge-balance-transition Janus hydrogels, and zwitterionic Janus patches illustrate different approaches for separating target-facing retention from non-target-facing antiadhesion or antifouling behavior [63,64,65,66]. These examples share a common design principle: spatial programmability is most valuable when the desired interface function is directional rather than uniform.

Spatial programming can also be achieved without a simple two-sided Janus architecture. Surface topology, microstructure, patterned chemistry, regional crosslinking, and spatially heterogeneous mechanics can localize adhesion, stiffness, permeability, degradation, or release within selected regions of the same hydrogel [43]. Engineered surface network topology has been used to program hydrogel adhesion in magnitude, spatial distribution, and kinetics without changing bulk hydrogel composition or mechanics [87]. In regenerative settings, macroporous hydrogels with spatiotemporally programmed mechanical properties illustrate how pore architecture, surface chemistry, stiffness, and degradation can be coordinated to guide stem-cell-assisted bone regeneration [84]. These examples indicate that spatial programmability may involve patterned mechanical support, localized permeability for tissue infiltration, region-specific degradation, or confined release of antimicrobial, anti-inflammatory, or pro-regenerative factors, not only adhesive-versus-antiadhesive surfaces.

The benefit of spatial programming depends on whether regional behavior is preserved after deployment. Janus or patterned materials may lose functional selectivity if they fold, rotate, migrate, swell asymmetrically, or expose an adhesive surface to non-target tissues [63,65]. This concern is especially relevant in wet and mobile operative fields, where blood, lavage fluid, organ motion, gravity, and limited visualization can alter material orientation or coverage after placement [20,58]. Temporal programming defines when material behavior should change, whereas spatial programming defines where those changes should occur. These principles are applied below across representative operative contexts before considering DNA-based hydrogels as molecularly programmable modules and the translational pathway required for clinical development.

## 5. Cross-Surgical Application Map

Programmable hydrogels become surgically relevant when the material program is matched to the dominant interface function, failure mode, and operative constraint of a specific application. A single operative field may contain several interface types, and the same material class may require different priorities depending on whether the dominant objective is sealing, separation, protection, or integration/reinforcement, and whether adjunct biological programs such as antimicrobial, immunomodulatory, angiogenic, or antitumor activity are required [23,24,25,26,33,35,39].

Representative applications, including lung surface air leaks, dural defects, gastrointestinal anastomoses, pericardial adhesion planes, repaired tendons, mesh interfaces, infected or impaired-repair operative beds, and selected tumor-resection cavities, therefore should not be grouped only by anatomy, specialty, stimulus type, or hydrogel chemistry. The relevant question is whether temporal sequencing, spatial asymmetry, stimulus responsiveness, or molecular recognition improves the operative task beyond what can be achieved by a static or otherwise similar nonprogrammed material.

For translational evaluation, two levels of comparison are needed. The first is a matched nonprogrammed analogue, which tests whether the programmed feature adds value to the underlying formulation. The second is the established clinical or preclinical benchmark for the intended use. Relevant benchmarks may include fibrin-based or synthetic sealants, low-swelling dural sealants, organ-specific adhesive patches, bioresorbable antiadhesion barriers, oxidized cellulose- or hyaluronate/carboxymethylcellulose-based materials, nerve or tendon wraps, unmodified or coated meshes, biologic or synthetic patches, and standard scaffold formulations [5,6,20,21,22,39,40,41,42].

### 5.1. Sealing and Containment Interfaces

Sealing interfaces arise when disrupted tissues must prevent the escape of gas, cerebrospinal fluid, luminal contents, digestive secretions, blood, or other biological fluids. Representative settings include lung surfaces after pulmonary resection, dural defects, gastrointestinal anastomoses or perforations, and ocular surface defects [23,24,25,50,55]. Although these anatomical contexts differ, they share several material requirements: rapid wet-tissue contact, interfacial adhesion, cohesive durability under pressure or motion, controlled swelling, resistance to washout, and degradation that does not outpace native repair [29,42,51,55]. A programmable sealing material is therefore best evaluated by whether its adhesion, mechanics, permeability, swelling, and degradation remain matched to the vulnerable sealing interval, rather than by gelation, adhesion strength, or burst pressure at a single time point alone.

The relevant sealing program is anatomy-dependent. Lung surfaces require materials that tolerate cyclic inflation, shear, and pressure gradients without behaving as rigid patches [23,42]. Elastic organ-adapted patches and lung-mimetic hydrofoam sealants illustrate how deformation tolerance, porous architecture, and lung-like viscoelastic behavior can be incorporated into pulmonary air-leak repair [42,88]. Dural interfaces require containment of cerebrospinal fluid while avoiding excessive swelling in confined cranial or spinal spaces [55,56]. Corneal repair adds optical clarity, surface regularity, and controlled in situ curing as additional constraints [50]. Gastrointestinal and other luminal interfaces impose chemical and microbiological challenges because the material may be exposed to pressure, mucus, digestive enzymes, bacteria, pH variation, and repetitive deformation [25,89,90,91,92].

For sealing applications, programmability is most compelling when a material transition addresses a time-dependent or site-specific failure risk. Examples include early adhesion followed by repair-matched degradation, low-swelling behavior in confined spaces, antimicrobial or anti-inflammatory release in contaminated luminal settings, or sensing of early leakage or rebleeding. Such claims require function-specific testing. Evaluation should include leak or burst pressure, leakage rate or volume, cyclic sealing stability, washout resistance, swelling or volumetric expansion, degradation profile, residual mechanical strength, local tissue response, and repair integrity under relevant wet and mechanically dynamic conditions [29,42,55,89,90,91,92].

### 5.2. Separation and Motion-Preserving Protection Interfaces

Separation and protection interfaces share a central problem: the material must control tissue contact without creating a new source of tethering, compression, friction, or impaired motion. Separation interfaces include peritoneal and pelvic surgery, pericardial and cardiac reoperations, visceral surface repair, intrauterine adhesion prevention, and implant-adjacent surfaces where contact with surrounding organs must be minimized [4,6,20,33]. Protection interfaces include peripheral nerves, tendons, ligaments, and other structures that must retain gliding, conduction, or repetitive motion during healing [21,22,35,36]. Unlike sealing, these applications do not primarily require pressure-driven containment. They require local retention, nonconstrictive coverage, low friction or antiadhesion behavior toward non-target tissues, and degradation after the vulnerable scar-forming period [20,32,33,34,35,36,37,38].

Peritoneal, pericardial, and visceral surfaces illustrate why separation often requires both spatial control and biological modulation. Conformal hydrogel barriers may reduce adhesion by combining physical coverage with retention, antifouling or antiadhesive surfaces, macrophage or inflammatory modulation, antioxidative activity, and controlled persistence during the adhesion-forming window [32,33,34,93]. Moving surfaces, such as the pericardium, add the requirement that the material remain locally retained without creating a new adhesion plane [20,94]. Visceral and gastrointestinal interfaces further emphasize the value of asymmetric or Janus designs, because the target-facing surface may need sufficient retention while the opposite surface should minimize adhesion, fouling, or tissue bridging toward adjacent organs [63,64,65,66,95]. The design question is therefore not only whether a barrier prevents adhesion, but whether it maintains directional behavior after deployment in a wet, mobile, and anatomically crowded operative field.

Nerve and tendon interfaces extend this principle from tissue-plane separation to functional protection. In these settings, the desired endpoint is preservation of gliding, conduction, or motion rather than barrier persistence alone [21,22,35,36]. Protective hydrogels may combine nonconstrictive coverage, lubricity, directional or two-sided adhesion, local regenerative or anti-inflammatory delivery, conductive or piezoelectric stimulation, ultrasound-responsive activation, or externally controlled behavior [37,38,75,76,96,97,98,99]. These added functions are valuable only if they preserve mobility without causing compression, tethering, swelling-related restriction, or persistent scarring. Evaluation should therefore prioritize surface-specific adhesion and antiadhesion, retention under motion, friction coefficient, tendon excursion, gliding resistance, range of motion, electrophysiological or motor recovery, pain-related behavior, histological scar tethering, and evidence of compression or constriction [35,36,37,38,75,76,96,97,98,99].

Electroactive and multifunctional hydrogel biointerfaces extend protection beyond passive wrapping by forming compliant electrical and mechanical bridges between neural tissue and implanted devices. Recent platforms combine conductivity with injectability, wet adhesion, anti-swelling behavior, antifouling properties, dimensional stability, or microscale device integration to maintain conformal tissue contact and reliable electrical coupling during recording or stimulation. An injectable conductive, adhesive, and anti-swelling hydrogel has enabled stable coupling to fine peripheral nerves and chronic vagus-nerve neuromodulation [74], whereas phase-transition-controlled hydrogel bioelectronics have supported miniaturized optical and electrical components for simultaneous neural stimulation and recording [79]. A non-deformable adhesive hydrogel patch has also been used to stabilize cortical microelectrocorticography devices while reducing interfacial displacement, fibrotic encapsulation, and glial recruitment during chronic recording [100]. These examples indicate that electroactive hydrogels should not be evaluated by bulk conductivity alone. Function-specific assessment should also include atraumatic fixation, dimensional and electrochemical stability, interfacial impedance, recording fidelity, stimulation efficiency, motion tolerance, and long-term tissue response.

### 5.3. Integration/Reinforcement Interfaces

Integration/reinforcement interfaces arise when an applied material must remain coupled to native tissue while providing mechanical support, biological incorporation, or both. Representative contexts include abdominal wall and pelvic floor mesh repair [39,40,41,101], soft-tissue or cardiovascular patches [42,102], and bone or hard-tissue regenerative scaffolds [84,85,103,104]. Unlike sealing or separation, early attachment is not sufficient to define success. A reinforcing material must maintain positional stability, transfer load without delamination or excessive stiffness mismatch, permit tissue ingrowth when intended, and avoid chronic inflammation, dense fibrotic encapsulation, seroma formation, shrinkage, or loss of mechanical support [39,40,41].

Programmability is relevant to integration/reinforcement because the desired interface state changes over time. Immediately after implantation, fixation, load sharing, and resistance to displacement may be the dominant priorities. During later healing, the material may need to permit cellular infiltration, vascularization, extracellular matrix deposition, degradation or remodeling, and mechanically compatible incorporation. A material that provides strong early fixation but blocks tissue ingrowth or provokes chronic fibrosis may fail as an integrating interface, whereas a highly degradable or compliant material may fail to support load transfer during the vulnerable early period.

Mesh-associated and patch-based interfaces illustrate the difficulty of balancing fixation, tissue ingrowth, antiadhesion behavior, and host response. Hydrogel coatings can modify early inflammation, bacterial interaction, collagen organization, macrophage phenotype, and visceral adhesion, but the relevant endpoint remains durable tissue–material coupling rather than bioactivity alone [39,40,41,101,105]. Cardiovascular, soft-tissue, bone, and hard-tissue interfaces impose additional requirements related to deformation tolerance, anisotropic or auxetic mechanics, fatigue resistance, pore architecture, degradation kinetics, mineralization, angiogenesis, and osteogenic cues [42,84,85,102,103,104]. For these applications, programmable behavior should be evaluated by whether it improves fixation, load transfer, tissue ingrowth, remodeling, or long-term mechanical support compared with nonprogrammed materials of similar baseline mechanics.

Recent multifunctional platforms extend this integration/reinforcement logic by coupling mechanical support with electrical conduction, spatial asymmetry, or time-dependent adaptation. Injectable conductive hydrogel electrodes delivered through the coronary venous system have been used to establish conduction pathways toward otherwise inaccessible myocardial regions and restore native-like pacing, illustrating that electroactivity becomes clinically relevant only when delivery, tissue coupling, electrical stability, and functional rhythm correction are demonstrated [106]. In dynamic soft-tissue repair, a super-structured porous hydrogel combined high early strength and fatigue resistance with a gradual reduction in mechanical properties during later healing, thereby maintaining initial support while reducing restriction of physiological motion. Its asymmetric porous surfaces simultaneously promoted tissue repair on one side and limited visceral adhesion on the other in a rabbit diaphragm-defect model [107]. These examples show that multifunctionality and adaptiveness are translationally meaningful when distinct material features resolve competing interface requirements across space or time. Evaluation should therefore include operative delivery, positional stability, fatigue durability, electrical or mechanical coupling, directional tissue response, remodeling, motion preservation, and comparison with simpler or established controls.

### 5.4. Adjunct Biological Programs Supporting Interface Control

Some operative sites require biological modulation in addition to mechanical interface control. In infected, diabetic, ischemic, contaminated, or recurrence-prone operative beds, programmable outputs such as antimicrobial release, biofilm control, reactive oxygen species (ROS) modulation, protease-responsive degradation, immune regulation, angiogenic support, or local surveillance may support protection, repair, integration, or local disease control [80,81,82,83,108,109,110,111,112,113,114,115]. These outputs should be treated as adjunct biological programs rather than additional principal interface functions. Their value depends on whether they improve local retention, infection control, inflammatory resolution, matrix deposition, residual disease control, or safe reoperation while preserving surgical usability.

These systems should be distinguished from conventional wound dressings, drug depots, or oncologic delivery platforms. A programmable hydrogel placed in a biologically active operative bed should still be evaluated through the interface it is intended to control. For infected or impaired-repair wounds, relevant endpoints may include material retention in an irregular bed, bacterial burden or biofilm formation, inflammatory profile, angiogenesis, matrix deposition, and compatibility with drainage, debridement, or reoperation. For selected tumor-resection cavities, local immunomodulation, phototherapy, checkpoint-related strategies, or recognition-based surveillance should be judged by whether they support local control without compromising tissue repair, adjuvant therapy, reoperation, or systemic safety [110,111,112,113,114,115]. Thus, evaluation should not rely only on wound closure, drug-release duration, tumor-size reduction, or signal generation, but should include interface-level performance, off-target activation, systemic toxicity, and compatibility with subsequent clinical management.

Across these applications, the same principle applies: the material program should be matched to the dominant interface objective and the most likely failure mode. Sealing interfaces prioritize containment under wet, pressurized, or cyclically deforming conditions. Separation and protection interfaces prioritize directional retention, low non-target adhesion, lubrication, preserved motion, and time-limited persistence. Integration/reinforcement interfaces require early fixation and load sharing followed by tissue ingrowth, remodeling, or durable coupling. Adjunct biological programs may address infection, inflammation, impaired repair, or residual disease, but they do not remove the need to demonstrate surgical usability, safety, and interface-level benefit. Table 4 provides a compact application map linking operative contexts to dominant interface functions, programmable design opportunities, and key evaluation priorities.

**Figure 3:**
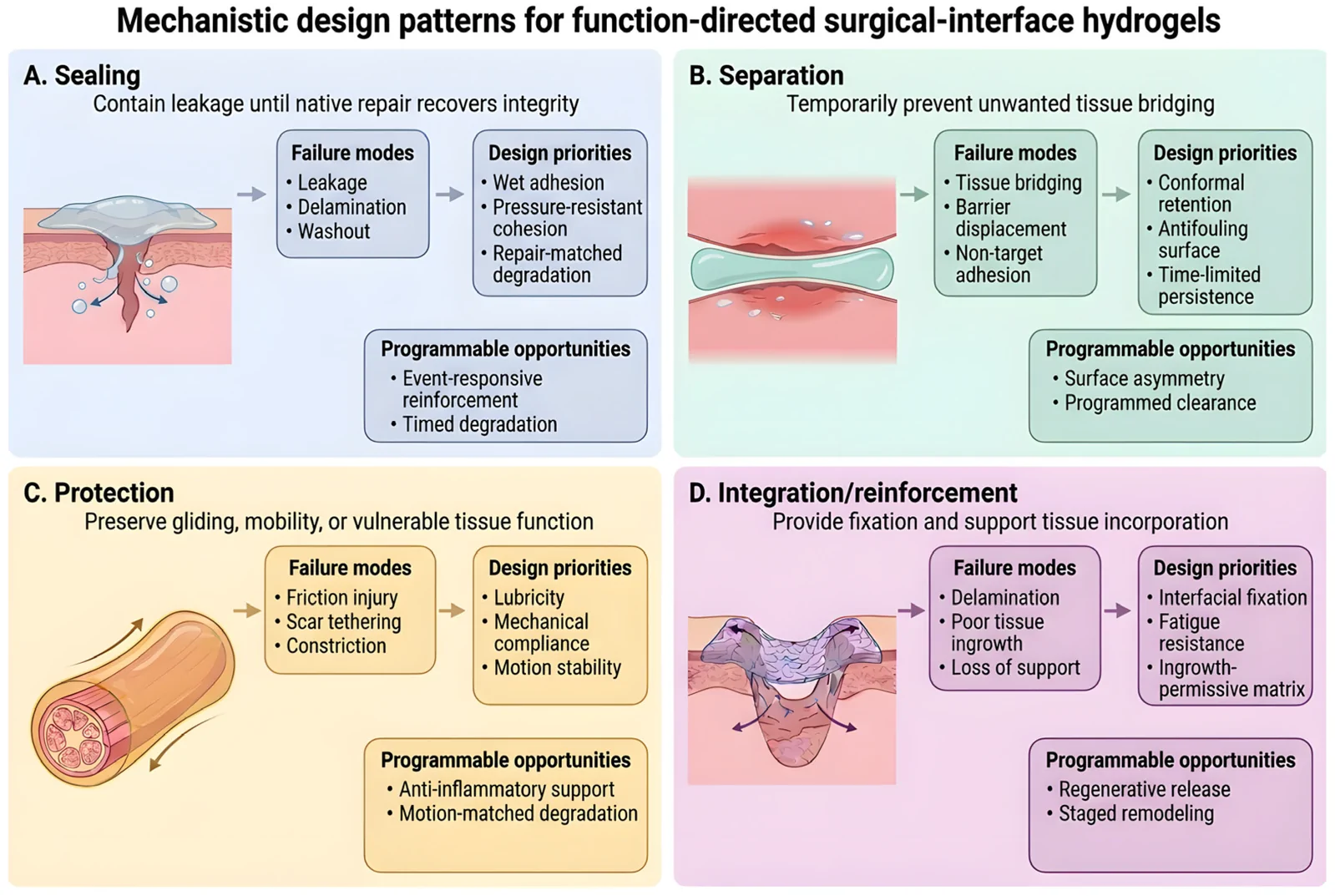
Mechanistic design patterns for function-directed surgical-interface hydrogels. Representative schematic summaries are shown for the four principal surgical-interface functions used throughout this review: (**A**) sealing, (**B**) separation, (**C**) protection, and (**D**) integration/reinforcement. For each function, the schematic links a representative surgical context with the dominant failure modes, major hydrogel design priorities, and selected programmable opportunities. Sealing emphasizes wet adhesion, cohesion, and repair-matched degradation to prevent leakage and washout. Separation emphasizes conformal retention, antifouling behavior, and programmed clearance to reduce unwanted tissue bridging. Protection emphasizes lubricity, compliance, and motion-matched degradation to preserve gliding and minimize friction-related injury or tethering. Integration/reinforcement emphasizes interfacial fixation, fatigue resistance, and ingrowth-permissive matrices to support tissue incorporation and sustained mechanical function. These representative schematics are intended to be illustrative rather than exhaustive and are included to improve the visual linkage between surgical failure modes and hydrogel design logic.

To improve the visual intuitiveness of the present framework, Figure 3 provides a schematic summary of representative mechanistic design patterns across the four principal surgical-interface functions discussed in this review: sealing, separation, protection, and integration/reinforcement. The figure links representative surgical contexts with dominant failure modes, key hydrogel design priorities, and selected programmable opportunities, thereby helping connect function-specific clinical problems with material-design logic.

Against this compact application map, DNA-based hydrogels merit separate consideration because they provide a molecularly explicit model of sequence-encoded recognition, assembly, degradation, release, and logic-like material behavior.

## 6. DNA-Based Hydrogels as Molecular Programmability Modules

DNA-based hydrogels occupy a distinct position in this framework because nucleic-acid sequences can couple molecular recognition to material behavior. This section is not intended to provide an exhaustive review of DNA hydrogels. Instead, DNA-based systems are used as a molecularly explicit example of how recognition, assembly, crosslinking, degradation, release, and logic-like behavior can be encoded into hydrogel materials and then evaluated through the surgical-interface framework developed above.

For surgical applications, the key question is not whether a hydrogel contains DNA, but whether the sequence-encoded module produces a useful material change at the operative boundary. Sequence-defined domains may act as structural elements, crosslinkers, degradable linkers, aptameric or enzymatic recognition modules, strand-displacement circuits, or CRISPR-responsive components [13,14,17,18,117]. DNA-based hydrogels are therefore best viewed as molecular programming modules or prototype programmable interfaces, rather than as immediate replacements for established sealants, barriers, or reinforcement materials.

This modular interpretation provides a practical way to connect DNA-based hydrogels with surgical-interface evaluation. A DNA-containing hydrogel should not be evaluated only by sequence programmability, gel formation, or trigger responsiveness. The relevant question is which DNA-encoded module is being used, what input it recognizes, what material transition it produces, which interface function that transition supports, and what nonprogrammed or sequence-specific control is needed to prove added value. Table 5 maps representative DNA-based molecular modules to encoded roles or outputs, surgical-interface relevance, and essential control and limitation considerations.

### 6.1. DNA as a Molecular Programming Language for Hydrogels

The programmability of DNA-based hydrogels originates from the ability of nucleic-acid sequences to function simultaneously as structural elements, recognition modules, degradable linkers, and information-bearing design codes. Complementary base pairing allows crosslinks, junctions, and recognition domains to be specified by sequence, whereas hybridization strength and exchange kinetics can be adjusted by strand length, guanine-cytosine (GC) content, mismatch design, sticky-end geometry, and toehold accessibility [13,14,117]. This direct relationship between molecular code and network behavior distinguishes DNA-based hydrogels from many stimuli-responsive polymer systems in which a responsive chemical motif is incorporated without an equally explicit correspondence between molecular information and material state.

DNA-based hydrogels can be constructed as pure DNA networks or as hybrid systems in which DNA domains are integrated into synthetic or natural polymer backbones. Pure DNA systems, including branched motifs, nanostars, Y-shaped scaffolds, rolling-circle-amplified strands, and complementary overhangs, demonstrate how self-assembly, pore structure, degradation, cargo retention, and network mechanics can be programmed through nucleic-acid architecture [117,118,119]. Hybrid systems use DNA as programmable crosslinkers, recognition elements, degradable linkers, or switchable modules while the non-DNA component provides mechanical strength, processability, adhesion, or structural stability [13,17,18]. This distinction is important for surgical translation. Pure DNA networks are valuable for demonstrating sequence-controlled behavior, whereas hybrid or composite designs are more likely to meet the mechanical and handling requirements of operative interfaces.

At the network scale, DNA-mediated crosslinking provides a mechanism for programming mechanics and remodeling. The lifetime, density, geometry, and reversibility of DNA crosslinks can modulate stiffness, stress relaxation, viscoelasticity, and on-demand matrix deconstruction without necessarily changing the underlying polymer concentration [13]. DNA nanostar systems similarly show that nucleic-acid architecture can influence three-dimensional network organization and bulk rheology [118]. DNA motifs can also encode stimulus-coupled changes in material state, as illustrated by stiffness-switchable DNA-based hydrogels and aptamer-containing systems in which target binding alters network mechanics [16,120]. These examples are relevant to surgical interfaces because many interface functions depend not only on initial modulus, adhesion, or degradation time, but also on how the material dissipates stress, remodels, and persists over time.

### 6.2. DNA Modules for Sensing, Actuation, Release, and Logic-like Behavior

A defining feature of DNA-based hydrogels is that molecular recognition can be coupled directly to a material output. The input may be a nucleic-acid sequence, protein, small molecule, enzyme, cell-surface marker, pathogen-associated signal, or disease-associated biochemical cue. Recognition may be encoded by complementary hybridization, aptamer binding, DNAzyme activity, CRISPR-associated nuclease activation, or strand-displacement reactions [15,18,121,122]. The output may include hydrogel degradation, sol–gel transition, stiffness change, altered permeability, fluorescence activation, cargo release, exposure of bioactive motifs, or loss of material function. This input–recognition–output logic makes DNA-based hydrogels useful prototypes for programmable surgical interfaces, in which a local event could be converted into diagnostic signaling, therapeutic release, degradation, permeability change, or another material-state transition.

CRISPR-responsive hydrogels illustrate how sequence-specific recognition can be amplified into material actuation. Cas12a-based systems can cleave DNA structural elements or DNA anchors after guide-defined target recognition, producing outputs such as hydrogel degradation, permeability change, signal generation, or cargo release [15,122]. These systems remain early-stage for surgical use, but they demonstrate how a user-defined molecular input could be translated into a material transition relevant to local sensing, release, or degradation.

Aptamer-containing DNA-based hydrogels provide a complementary route for converting molecular recognition into material output. Aptamers can bind proteins, metabolites, ions, or cell-surface markers and thereby alter crosslinking, swelling, fluorescence, release, or local retention [17,18,19,121]. DNAzymes and strand-displacement circuits can further introduce catalytic or logic-like behavior, allowing material transitions to depend on sequence recognition, competing strands, or combinations of inputs [15,18,121]. This logic-like behavior is relevant because postoperative environments are heterogeneous and noisy. Blood, lavage fluid, inflammatory mediators, proteases, necrotic tissue, bacteria, and implanted materials may all generate overlapping biochemical signals. A clinically useful programmable interface would therefore need to distinguish a true target event from background activation. Appropriate controls include recognition-deficient, trigger-insensitive, non-cleavable, or nonprogrammed formulations to determine whether the DNA-encoded recognition element itself improves sensing, release, degradation, or therapeutic timing.

### 6.3. Mapping DNA-Based Hydrogel Behaviors to Surgical-Interface Functions

When viewed through the surgical-interface framework, DNA-based hydrogels should be interpreted according to the interface function they support rather than by nucleic-acid chemistry alone. Current evidence is uneven across functions. Hemostatic and hemorrhage-responsive systems provide the most direct link to immediate operative events [19,70], whereas wound and infected-wound models provide useful analogues for temporal therapeutic programming, local biological modulation, and protection of impaired repair environments [124,125,126]. Tumor-bed systems illustrate postoperative sensing and response within biologically active operative beds [113], and bone-regeneration models support the idea that DNA networks can instruct tissue integration, although mechanical reinforcement remains a separate requirement [103]. In contrast, direct evidence remains limited for DNA-based hydrogels as antiadhesion barriers, nerve wraps, tendon-gliding interfaces, or durable structural reinforcements.

Sealing and hemostasis are the most immediate interface functions to which DNA-based hydrogels have been applied. Hemostatic DNA hydrogels and hemorrhage-responsive implantable systems illustrate two early routes for interface control: direct local stabilization and event-triggered sensing or therapeutic release [19,70]. However, hemostasis is not identical to sealing of air, cerebrospinal-fluid, or gastrointestinal defects, and these systems still require function-specific testing for retention, washout, pressure resistance, swelling, tissue response, and repair integrity.

DNA-based hydrogels have also been used to modulate biologically active wound, tumor-bed, and regenerative interfaces. These examples are best interpreted as temporal programming or biological-instruction analogues rather than as direct evidence for intraoperative sealants, antiadhesion barriers, nerve wraps, or tendon-gliding interfaces. For integration/reinforcement, DNA modules are more compelling as instructional or remodeling elements than as load-bearing matrices; they are most likely to require incorporation into mechanically stronger hydrogel, polymer, mineralized, or composite scaffolds [13,17,18,103,117]. Accordingly, DNA-based surgical-interface claims should be framed according to the function actually tested: hemostatic stabilization, event-triggered sensing or release, biologically active repair support, remodeling instruction, or conceptual antiadhesion/protection programming.

### 6.4. Design Rules for DNA-Based Surgical-Interface Modules

Several design principles follow from mapping DNA-based hydrogels to surgical-interface functions. First, the DNA module should have a defined interface task. Sequence programmability is valuable only if the encoded behavior supports a specific outcome, such as local sensing of rebleeding, triggered release of a hemostatic or antimicrobial payload, degradation after an adhesion-forming window, or remodeling coupled to tissue ingrowth. A DNA-containing material should not be described as programmable solely because it contains hybridizable or degradable strands [10,13,45,117].

Second, the DNA module should be paired with an appropriate structural matrix. For many surgical applications, the non-DNA component will need to provide wet-tissue adhesion, cohesive strength, elasticity, fatigue resistance, swelling control, injectability, sprayability, or minimally invasive deliverability. The DNA component can then provide recognition, logic-like activation, release control, degradation timing, or biological instruction. This division of labor may be the most realistic near-term strategy for translating DNA programmability into surgical-interface materials [13,17,18,117].

Third, the programmed transition should remain predictable under operative conditions. DNA hybridization, aptamer binding, strand displacement, and nuclease-mediated degradation can be altered by salts, serum proteins, extracellular nucleases, blood, pH, oxidative stress, temperature, lavage fluid, tissue debris, and mechanical deformation [13,14,17,18,117,121]. These factors may change the threshold, speed, magnitude, or specificity of the encoded response. Surgical testing should therefore include relevant biological fluids, tissue contact, irrigation, motion, and off-target biochemical environments rather than relying only on buffered in vitro conditions.

Fourth, DNA-based programmability should be evaluated against nonprogrammed controls. Appropriate comparators include DNA-free hydrogels with similar baseline properties, scrambled-sequence controls, non-cleavable linkers, recognition-deficient aptamers, trigger-insensitive formulations, or materials with the same payload delivered without programmed release. These controls are necessary to determine whether the DNA-encoded module provides a functional advantage beyond the underlying hydrogel composition, drug loading, or degradation profile [10,45].

### 6.5. Translational Limits of DNA-Based Surgical Hydrogels

The same molecular features that make DNA-based hydrogels programmable also create translational barriers when they are placed in operative environments. Many DNA-rich networks are soft, weak, highly hydrated, or nuclease-sensitive, and may not tolerate pressure gradients, cyclic deformation, shear, traction, blood exposure, lavage, or surgical handling without reinforcement [17,18,117]. Nuclease sensitivity may be useful when degradation is intentionally programmed, but uncontrolled degradation could eliminate sensing, release, adhesion, or structural function before the relevant interface task is complete. Stabilizing modifications, protective formulations, hybrid matrices, or encapsulation strategies may improve persistence, but can also alter biocompatibility, degradation, cost, and manufacturing complexity [17,18,127,128,129].

Biological safety and product handling are equally important. DNA motifs, degradation fragments, nanoparticles, cationic carriers, or associated delivery systems may influence innate immune activation, inflammation, coagulation, or local tissue response [17,18,123]. DNA-based systems may also be sensitive to heat, ionizing radiation, hydrolysis, nuclease contamination, pH, ionic strength, drying, freezing, or prolonged storage [17,18,117,130]. Because material behavior may depend on sequence purity, strand length, hybridization efficiency, strand stoichiometry, folded structure, residual reagents, endotoxin burden, and payload loading, sterilization, storage, and batch reproducibility should be treated as part of functional validation rather than downstream product handling alone.

Regulatory evaluation may be more complex than for conventional hydrogels because a DNA-based surgical-interface material may function as a device, drug-delivery system, biologic, diagnostic sensor, or combination product depending on its primary mode of action [131,132]. The translational pathway should therefore define the primary clinical claim early and align testing with that claim. Overall, the near-term role of DNA-based hydrogels is likely to lie less in replacing established sealants, barriers, or reinforcement materials than in providing programmable recognition, sensing, release, degradation, or remodeling modules within mechanically robust and surgically usable hybrid systems.

## 7. Translational Evaluation Pathway

Programmable surgical-interface hydrogels require a translational pathway that links encoded material behavior to procedure-specific performance. Demonstrating stimulus-triggered swelling, degradation, release, adhesion, fluorescence, or mechanical change is insufficient unless the programmed behavior improves a function-specific interface outcome under relevant biological, mechanical, and operative conditions [10,11,45]. Translation should therefore proceed from molecular or material proof-of-concept toward progressively more realistic models of interface performance, consistent with staged approaches to surgical and device innovation [133].

**Figure 4:**
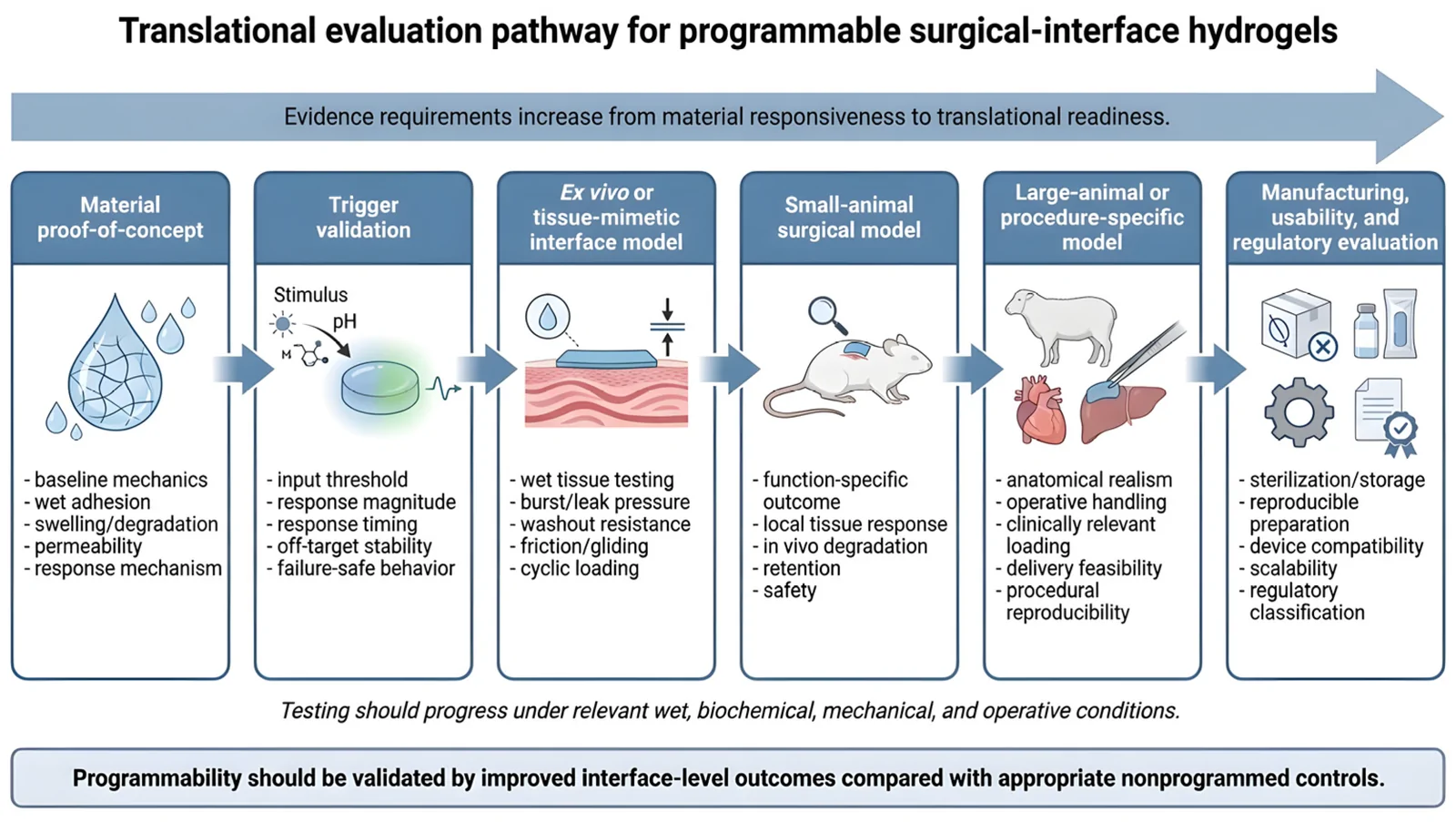
Translational evaluation pathway for programmable surgical-interface hydrogels. Evaluation should progress from material proof-of-concept and trigger validation to ex vivo or tissue-mimetic interface testing, small-animal surgical models, large-animal or procedure-specific operative models, and human feasibility or comparative studies when appropriate. Across stages, programmed behavior should be tested for input relevance, off-target stability, function-specific interface performance, operative usability, manufacturing and sterilization compatibility, biological safety, and regulatory positioning. Claims of programmability, surgical-interface efficacy, and translational readiness should be matched to the level of supporting evidence.

The relevant translational question is whether the encoded input–transition–output relationship remains reliable during preparation, delivery, activation, implantation, degradation, and tissue repair. This requirement applies across sealing, separation, protection, and integration/reinforcement, as well as to operative beds in which programmed biological outputs support infection control, inflammation resolution, angiogenesis, immune modulation, or local disease surveillance. Figure 4 summarizes this staged translational evaluation pathway.

### 7.1. Evidence Ladder and Minimum Evaluation Set

Evidence for programmable surgical-interface hydrogels should be stratified according to how directly it supports a function-specific operative claim. Material studies can establish that an encoded trigger–response relationship or spatially asymmetric behavior exists [10,45,59,63,64,65,66,87,134,135], and DNA sequence-specific actuation provides a molecularly explicit example of this logic [15,122]. These studies are necessary for mechanism definition, but they do not by themselves establish surgical relevance. A material that performs under idealized buffer conditions may fail when exposed to blood, lavage fluid, tissue enzymes, cyclic motion, bacterial contamination, or competing biological signals.

The next level should connect the programmed transition to a tissue-facing interface model. Ex vivo, organ-on-bench, or tissue-mimicking systems can test whether the material retains function under wet contact, deformation, fluid flow, pressure gradients, friction, or biological contamination. Relevant endpoints should be selected according to the intended interface function: leak or burst pressure and cyclic sealing stability for sealing; target coverage, directional antiadhesion, and tissue bridging for separation; friction, gliding resistance, motion preservation, and constriction risk for protection; and fixation strength, fatigue behavior, tissue-ingrowth permissiveness, and residual load transfer for integration/reinforcement. Standardized mechanical tests, including burst, wound-closure, and lap-shear strength assays, can support selected sealing or adhesion claims [136,137,138], but programmable surgical-interface claims should also include appropriate nonresponsive or nonprogrammed controls with similar baseline properties [10,11,45].

In vivo evidence should then address whether the material performs in a biologically active and procedurally realistic setting. Small-animal models can provide early information on biocompatibility, degradation, local inflammation, infection control, tissue repair, or proof of function, but they often do not capture the tissue thickness, pressure gradients, organ motion, access constraints, fluid exposure, or operative handling encountered in human surgery. Large-animal or procedure-specific models may be necessary when the intended claim involves air or fluid sealing, confined-space swelling risk, minimally invasive delivery, cyclic loading, tendon or nerve gliding, mesh-like reinforcement, or reoperation [133,139,140].

For implantable surgical applications, early biocompatibility and single-time-point efficacy are insufficient. Chronic stability should be evaluated over a duration that encompasses the intended functional lifetime of the material, the major phase of degradation, and the transition from material-provided support to native tissue repair. Longitudinal assessment should include implant position and dimensional stability, interfacial adhesion and cohesion, delamination, swelling, mass loss, changes in mechanical or electrical function, release of degradation products, and preservation of any programmed response threshold, spatial asymmetry, or sequential behavior. Mechanical testing should reproduce the relevant strain amplitude, pressure cycle, loading frequency, and cumulative cycle number because progressive crack growth, fatigue damage, or interfacial failure may occur despite acceptable initial strength [42,52,100,107]. Accelerated degradation and fatigue studies can support material screening and mechanism definition, but they should be correlated with real-time and in vivo observations rather than treated as substitutes for chronic implant evaluation.

Host response and tissue remodeling should likewise be evaluated as evolving interface outcomes rather than as isolated histological observations at a single explant time point. Assessment should distinguish resolving postoperative inflammation from persistent macrophage and foreign-body giant-cell responses, dense fibrotic encapsulation, disorganized collagen deposition, seroma formation, calcification or mineralization when relevant, and injury to adjacent tissues. Serial imaging or functional testing combined with predefined early, intermediate, and late explant time points can determine whether degradation remains synchronized with tissue repair and whether declining material support is replaced by stable tissue ingrowth, vascularization, extracellular matrix deposition, and native load transfer. The desired long-term outcome remains function-dependent: a sealant, barrier, or protective wrap should resolve without chronic compression, tethering, or unwanted adhesion, whereas a reinforcing scaffold should maintain early fixation and later permit mechanically compatible incorporation and remodeling [39,40,41,84,85,86,101,107]. Chronic studies should therefore compare the programmable material with both a matched nonprogrammed control and an established implant benchmark and should report late failure modes rather than early success alone.

A practical evidence ladder should therefore progress through linked stages: mechanism-confirming material studies, trigger validation under relevant biological and off-target conditions, ex vivo or tissue-mimicking interface tests, small-animal surgical models, large-animal or anatomically realistic operative models, and human feasibility or comparative studies when appropriate. Table 6 translates this evidence ladder into a minimum evidence set for programmable surgical-interface hydrogels. It should be interpreted as complementary to Table 2: Table 2 defines the function-specific design target, whereas Table 6 defines the evidence needed to claim that a programmed material achieves that target.

### 7.2. Operative Usability and Failure-Safe Testing

Operative usability is a translational determinant, not a secondary engineering detail. A programmable hydrogel may show precise input–output behavior under controlled laboratory conditions but fail clinically if it requires complex preparation, has an impractical working time, clogs the applicator, runs off before fixation, obscures the operative field, cannot be delivered through the intended access route, or cannot be oriented correctly [46,58]. Preparation or deployment errors may also alter the encoded response itself; incorrect mixing, incomplete curing, wrong orientation of an asymmetric material, mistimed activation, or trigger diffusion beyond the target field may change adhesion, degradation, release, sensing, or spatial selectivity.

Usability testing should therefore approximate the intended surgical workflow, including preparation time, number of user steps, applicator compatibility, working and curing windows, field visibility, deployment accuracy, tolerance to blood or irrigation fluid, and operator-to-operator variability [46,58,141,142]. Minimally invasive use adds constraints related to catheter or trocar compatibility, access angle, visualization, delivery distance, and limited ability to reposition the material after deployment. For spatially programmed hydrogels, testing should confirm that the operator can identify, orient, deploy, and maintain the target-facing and non-target-facing surfaces correctly [63,64,65,66,87].

Failure-safe behavior should be evaluated alongside intended performance. Programmable hydrogels may introduce failure modes that are less relevant to static materials, including off-target activation, incomplete activation, premature or delayed degradation, unintended cargo release, excessive swelling after activation, loss of surface asymmetry, or persistence of a biologically active component after the interface function is no longer needed. A clinically credible programmable interface should define both the intended program and the acceptable boundaries of failure, such as maximum allowable swelling, minimum retention time, safe degradation window, acceptable off-target activation threshold, limits on heat or ultrasound exposure, absence of constrictive behavior, and removability after misapplication [143].

### 7.3. Manufacturing Scale-Up, GMP, Sterilization, Storage, and Regulatory Positioning

Manufacturing and sterilization are translation-critical requirements for programmable surgical-interface hydrogels because the encoded material behavior must survive production, storage, transport, preparation, and intraoperative deployment. For hydrogel products, translational control should include polymer composition, crosslinking density, precursor concentration, residual reagents, sterility, endotoxin burden, swelling, degradation, mechanical properties, and batch-to-batch reproducibility [130]. For programmable systems, these requirements extend to preservation of the programmed feature itself, such as enzyme-sensitive linkages, dynamic bonds, spatial asymmetry, trigger-responsive motifs, drug-loading profiles, or nucleic-acid-based recognition elements. A material should therefore not be characterized only immediately after synthesis. Its input sensitivity, response threshold, release profile, adhesion, degradation, and mechanical performance should be reassessed after sterilization, storage, reconstitution, and delivery.

Manufacturing scale-up should be treated as controlled process development rather than as the simple enlargement of a laboratory batch. Changes in mixing conditions, shear history, component-addition sequence, processing time and temperature, heat and mass transfer, reactor and filling geometry, and curing volume may alter network homogeneity, gelation kinetics, crosslink density, rheological behavior, payload distribution, and final hydrogel performance [149,150]. GMP-compatible development should therefore define raw-material specifications, critical material attributes, critical process parameters, in-process controls, acceptance criteria, validated analytical methods, lot-release testing, and change-control procedures for the final-use formulation and delivery system [144,148]. For multicomponent, drug-loaded, nucleic-acid-containing, or spatially patterned hydrogels, scale-up should additionally control component stoichiometry, sequence or payload integrity, residual reagents, endotoxin burden, fill volume, container–closure interactions, applicator performance, and preservation of the intended spatial or programmed behavior [149,150]. The applicable quality framework will depend on product identity and primary mode of action and may involve medical-device quality-system requirements, pharmaceutical or biologic GMP, or an integrated combination-product approach [145].

Sterilization strategy should be selected according to both the hydrogel platform and the programmed function. Terminal sterilization and aseptic processing should be evaluated using the final formulation, packaging configuration, and delivery system because heat, ionizing radiation, ethylene oxide, filtration, lyophilization, drying, or precursor-based storage may alter polymer molecular weight, crosslinking, residual chemistry, water content, phase behavior, pore structure, swelling, degradation, mechanical integrity, bioactivity, and trigger responsiveness [130,154]. These effects are formulation- and process-specific. In gelatin methacryloyl hydrogels, autoclaving and ethylene oxide treatment decreased stiffness, whereas gamma irradiation increased stiffness, reduced pore size, slowed degradation, and impaired sol–gel transition and printability [154]. In a Poloxamer 407 formulation, heat treatment altered gelation and structural properties, gamma irradiation caused oxidative degradation and loss of biocompatibility, whereas electron-beam irradiation better preserved the intended rheological and gelling behavior [152]. These examples demonstrate that sterilization cannot be selected solely on the basis of microbial inactivation.

Sterilization validation should therefore assess the relevant critical quality attributes before and after processing, including gelation or curing time, rheology, adhesion and cohesion, swelling, degradation, payload or sequence integrity, release profile, response threshold, spatial asymmetry, and applicator performance. Sterilization dose, temperature, exposure time, processing stage, packaging atmosphere, residual sterilant or degradation products, and container–closure interactions should be defined when applicable. For programmable systems, functional testing should be repeated after sterilization and after accelerated and real-time storage because a product may retain sterility and bulk composition while losing surface selectivity, enzymatic responsiveness, molecular recognition, or the intended activation threshold. Sterilization and storage should therefore be treated as part of final-product functional validation rather than as downstream packaging steps.

Regulatory positioning will depend on the intended primary mode of action, the active components included in the hydrogel, and whether the product raises device, drug, biologic, or combination-product considerations [131,132]. A hydrogel used primarily as a physical sealant, barrier, scaffold, coating, or reinforcement material may follow a device-oriented pathway, whereas systems that deliver drugs, biologics, cells, immunomodulators, diagnostic signals, or nucleic-acid components may raise combination-product considerations [131]. Biological evaluation should be planned according to the nature and duration of tissue contact, degradation products, local and systemic exposure, and the full set of material constituents rather than the hydrogel backbone alone [146,147]. Programmable hydrogels add an additional issue: the safety profile may differ before activation, during the programmed transition, and after degradation or payload release. Regulatory and preclinical evaluation should therefore address the material in each relevant state, including baseline, activated, partially activated, degraded, and off-target-exposed conditions.

Combination-product status should be determined from the final product configuration and intended clinical use rather than from hydrogel chemistry alone. A programmable hydrogel may constitute a single-entity combination product when a structural matrix and a drug, biologic, cell-based, or diagnostic constituent are integrated into one product; a co-packaged combination product when the hydrogel and a distinct delivery or activation component are supplied together; or a cross-labeled combination product when separately supplied products must be used together to achieve the intended effect [131]. In the United States, assignment of a combination product to a lead review center is generally based on its primary mode of action, and a pre-Request for Designation or formal Request for Designation may be used when product identity or jurisdiction is uncertain [132]. Classification influences the lead regulatory pathway but does not eliminate requirements applicable to the individual constituent parts. Accordingly, product developers should define the constituent parts, primary mode of action, activation or delivery device, intended claims, and final packaging configuration early, because changes in payload, trigger mechanism, applicator, or labeling may alter the regulatory strategy, manufacturing controls, human-factors program, and supporting preclinical or clinical evidence [131,132,145].

Regulatory examples in this review are intended to illustrate the type of evidence and product-definition logic required for surgical-interface materials rather than to prescribe a jurisdiction-specific pathway. Specific classification, preclinical testing expectations, and clinical evidence requirements may differ across regulatory systems, including FDA, EU MDR, and other national or regional frameworks. Nevertheless, the underlying translational questions remain similar: what is the primary mode of action, what components create the programmed behavior, what risks arise before and after activation or degradation, and what evidence is needed to support the intended clinical claim?

For translational development, the chemistry, manufacturing, and control package should be aligned with the claimed interface function and final-use product expectations [131,132,148]. Critical quality attributes should include the properties that determine the intended function: adhesion, cohesion, swelling, degradation, and leak resistance for sealing; surface selectivity, antiadhesion, lubricity, degradation, and absence of constrictive swelling for separation or protection; and mechanical support, degradation kinetics, tissue-ingrowth permissiveness, and host-response profile for integration/reinforcement. For DNA- or oligonucleotide-containing systems, additional control of sequence purity, strand length, amplification products, crosslinking ratio, residual reagents, endotoxin burden, formulation composition, ionic conditions, and container or delivery format may be required [127,128,129]. These considerations reinforce a central translational point: the programmed feature must remain reproducible and useful in the final surgical-use form.

### 7.4. Cost and Commercialization Barriers

Cost should be considered at both the development and per-procedure levels. Development costs arise from process scale-up, GMP-compatible production, sterilization and shelf-life validation, specialized analytical testing, regulatory studies, clinical evaluation, manufacturing infrastructure, packaging, and validation of the associated delivery device. Per-procedure costs may include the hydrogel formulation, single-use applicators or activation equipment, controlled storage or transport, preparation time, staff training, and additional operative steps. These burdens may increase when a platform contains drugs, biologics, cells, nucleic-acid modules, nanoparticles, spatially patterned components, or externally activated functions, because each added constituent or device component may require separate sourcing, characterization, stability control, and quality documentation [116,149,150,153].

Commercial viability therefore depends on whether the programmed function provides sufficient clinical or workflow value to justify this added complexity. A programmable hydrogel may demonstrate superior molecular precision but remain commercially unattractive if it has a short shelf life, depends on costly or variable raw materials, requires cold-chain distribution, involves multiple preparation steps, uses a proprietary applicator, or offers only a small improvement over an established sealant, barrier, patch, or scaffold. Early development should consequently define a target product profile that includes cost of goods, scalable sourcing, packaging and distribution, shelf life, procedural compatibility, training requirements, intellectual-property position, reimbursement or hospital-purchasing considerations, and the expected clinical advantage over current care. Commercialization should be judged not by material sophistication alone, but by whether the final product can be manufactured reproducibly, supplied reliably, integrated into surgical workflow, and adopted at a cost proportionate to its improvement in a defined interface-level outcome [149,150,151,153].

Clinically available sealants and antiadhesion barriers provide essential translational benchmarks for programmable surgical-interface hydrogels. Their established strengths, including standardized manufacturing, validated sterilization and shelf life, defined regulatory pathways, and familiar operative workflows, should not be discounted. At the same time, many current products exhibit largely predefined adhesion, mechanics, swelling, persistence, or barrier behavior and therefore cannot adjust to changing spatial or temporal requirements after deployment. Table 7 provides a concise, non-exhaustive comparison of representative clinically available material classes with the potential advantages, limitations, and evidence requirements of programmable hydrogel strategies. Product availability and approved indications vary by jurisdiction.

### 7.5. Defining the Level of Evidence and Claim

A recurring problem in the programmable hydrogel literature is that different levels of evidence are sometimes described using the same language, even though material programmability, interface-level performance, translational readiness, and clinical use represent different evidentiary claims [10,11,45,133,143]. A material may be stimulus-responsive without being programmable, programmable without being surgically useful, effective in a simplified animal model without being procedurally translatable, and translationally plausible without being clinically ready. These distinctions should be explicit because overclaiming at any stage can obscure the actual gap between molecular design, operative performance, and clinical adoption.

A molecular programmability claim is supported when the input, transition, and output are intentionally encoded, reproducible, and controllable compared with appropriate nonprogrammed controls. An interface-function claim is supported when that transition improves a defined surgical endpoint, such as leak resistance, reduced tissue bridging, preserved gliding, tissue ingrowth, mechanical support, infection control, or local disease surveillance. A translational-readiness claim requires additional evidence that the material can be manufactured reproducibly, sterilized or aseptically processed, stored, prepared, delivered, activated, and used safely within the intended operative workflow. A clinical claim requires procedure-specific evidence against existing standard materials or clinically relevant alternatives.

This distinction is particularly important for multifunctional systems. A hydrogel that adheres, releases a drug, degrades in response to enzymes, and contains a diagnostic module may appear sophisticated, but its translational value depends on whether each function is necessary, coordinated, and superior for the intended surgical-interface objective. The goal is not to maximize the number of responsive features, but to encode the minimum material program required to improve a defined interface outcome. Clear claim-level language will make programmable surgical-interface hydrogels more interpretable, more comparable across studies, and more credible for clinical translation.

## 8. Future Perspectives

Future progress in programmable surgical-interface hydrogels will depend on moving from material-centered innovation toward function-defined interface design. The field already contains diverse responsive chemistries, adhesive mechanisms, degradation motifs, spatially asymmetric architectures, and DNA-based recognition systems. The next challenge is to organize these capabilities into design rules that begin with a surgical failure mode and work backward to the minimum material program required to address it. A sealing interface should not be designed only for maximum adhesion, but also for wet-field attachment, cyclic stability, swelling tolerance, repair-matched degradation, and failure-safe behavior. Separation and protection interfaces should prioritize directional surface behavior, antiadhesion or lubrication toward non-target tissues, preservation of motion, and time-limited persistence. Integration/reinforcement interfaces should prioritize fixation, load transfer, mechanical compatibility, tissue ingrowth when intended, and host-response control.

Data-driven and computational approaches may help build these function-specific design rules, but only if material descriptors are linked to operative and interface-level outputs. Machine learning, computational hydrogel design, molecular simulation, finite-element-inspired modeling, and artificial intelligence may accelerate material discovery when formulation variables are connected to leak pressure, cyclic sealing stability, directional adhesion, gliding resistance, material retention, degradation timing, tissue-ingrowth depth, host-response profile, or local recurrence control [157,158,159]. Useful datasets should also capture operative descriptors such as tissue type, fluid exposure, motion, defect geometry, access route, activation method, and dominant failure mode. Negative or failed designs should be included because delamination, washout, swelling-related compression, off-target adhesion, loss of gliding, premature degradation, and poor tissue ingrowth may be more informative for surgical-interface design than optimized material performance alone.

A second direction is the development of feedback-informed and modular interface materials. Many current responsive hydrogels remain open-loop systems: they change in response to a cue, but the response is not calibrated to the evolving state of the interface. Future systems may integrate sensing, actuation, release, degradation, and feedback-like behavior within one material platform. Electroactive and bioelectronic hydrogels represent a particularly relevant extension of this concept because compliant conductive interfaces can combine tissue coupling, physiological recording or stimulation, sensing, and adaptive actuation within a surgically deployable platform. Their value, however, should be judged by interface-level improvements, including atraumatic fixation, stable electrical impedance, motion-tolerant signal fidelity, effective stimulation, and preservation or restoration of physiological function, rather than by conductivity or multifunctional integration alone [74,79,100].

This convergence may extend across implantable bioelectronics, wearable systems, and soft robotic devices. In implantable systems, hydrogels can provide mechanically compliant, ionically or electronically conductive, and biologically interactive interfaces between tissues and electrodes or other device components [160]. Wearable hydrogel platforms may serve as external companion systems for continuous peri-incisional or rehabilitation-related monitoring of strain, pressure, temperature, electrophysiological activity, or biochemical signals [161], whereas hydrogel-based soft robotic systems may enable conformal actuation, programmed shape change, and mechanically adaptive assistance [162]. More advanced platforms may combine these capabilities within closed-loop architectures that link physiological sensing to a defined decision rule and therapeutic actuator, allowing electrical stimulation, molecular release, or mechanical actuation to be adjusted according to the evolving state of the interface rather than delivered according to a fixed schedule [163]. However, the coexistence of sensing and actuation should not by itself be described as closed-loop therapy. Validation should define the monitored variable, sensing accuracy, controller or material logic, response latency, therapeutic range, power and communication requirements, stability under hydration and motion, fail-safe state, and the respective roles of clinician and patient oversight [160,163]. For surgical-interface applications, the additional electronic or robotic complexity is justified only when the integrated platform improves a procedure-specific endpoint compared with a simpler open-loop or nonprogrammed material.

DNA and nucleic-acid hydrogel systems are relevant to this direction because molecular recognition, strand displacement, aptamer binding, DNAzyme reactions, and CRISPR-associated cleavage can be linked to degradation, permeability change, cargo release, or signal generation [15,19,117,121,122,164]. However, a sensing event is clinically meaningful only if it changes management, improves local therapy, or prevents an interface-level failure. Similarly, feedback-like behavior is useful only if it improves a predefined surgical endpoint compared with a simpler nonprogrammed material.

Hybrid and modular architectures are likely to be more translationally realistic than purely molecularly programmed hydrogels. DNA components, aptamers, enzyme-sensitive domains, dynamic bonds, nanoparticles, or responsive release modules may provide recognition or information-processing functions [13,117,164], whereas mechanically stronger polymer networks, fiber reinforcement, tough adhesive layers, or composite structures may provide operative durability [42,53]. Future systems should therefore separate and validate these roles deliberately. The strongest designs will not necessarily be the most complex, but those in which each module has a necessary and testable role in the intended interface function.

Finally, programmable hydrogel development should become procedure-specific from the earliest design stage. Interfaces differ by organ system, access route, defect geometry, tissue quality, contamination risk, patient comorbidity, and expected healing trajectory. Image-informed design, computational modeling, and additive manufacturing may eventually help tailor hydrogel geometry, stiffness gradients, release profiles, or surface orientation to defined operative contexts [158,159]. However, personalization should not increase complexity beyond what can be manufactured, sterilized, delivered, and evaluated reproducibly. The field will advance most effectively when programmability is treated not as a generic smart-material feature, but as a constrained design strategy for solving a specific surgical-interface failure mode.

## 9. Conclusions

Programmable hydrogels for surgical use should be evaluated as interface-directed materials rather than as stimulus-responsive materials in general. The relevant question is not whether a hydrogel responds to a generic biochemical or physical cue, but whether the encoded transition improves a defined surgical-interface function under procedure-relevant conditions. Sealing, separation, protection, and integration/reinforcement provide a practical framework for connecting hydrogel design to operative failure modes, including leakage, unwanted adhesion, scar-related tethering, poor tissue incorporation, delamination, loss of support, and maladaptive host response. Hemostatic, antimicrobial, immunomodulatory, angiogenic, or recurrence-related outputs are most useful when they support one of these interface objectives and are tested against appropriate nonresponsive, nonprogrammed, or clinical benchmark controls.

DNA-based hydrogels illustrate the promise and limitations of molecular programmability. Nucleic-acid sequences can encode recognition, assembly, crosslinking, actuation, degradation, release, and logic-like behavior, but their near-term surgical value is likely to depend on incorporation into mechanically robust and surgically usable hybrid systems rather than on stand-alone DNA hydrogel platforms. Future studies should therefore make programmability claims necessary, testable, reproducible, and superior for a specified interface function, with evidence extending from encoded material behavior to operative handling, sterilization, storage, safety, and procedure-specific performance.

## Figures and Tables

**Table 1:** Comparative positioning of representative hydrogel review frameworks in relation to surgical-interface control.

Review Tradition and Representative References	Primary Organizing Axis	Principal Contribution	Recurring Limitation for Cross-Specialty Surgical-Interface Design	Extension Provided by the Present Review
Broad hydrogel-platform reviews [7,8,9]	Polymer source or backbone, network architecture, crosslinking, delivery format, material properties, and biomedical applications	Broad comparison of hydrogel platforms and chemistry–property–application relationships	The operative failure mode, required interface function, and function-specific comparator or endpoint generally remain implicit	Begins with the surgical boundary and failure mechanism before selecting material properties or chemistry
Programmable or stimuli-responsive hydrogel reviews [10,12]	Biological, chemical, or physical inputs; material transitions; dynamic behavior; broad biomedical applications	Establishes conceptual distinctions among responsive, dynamic, and programmable material behaviors	Demonstration of a material-state change does not necessarily establish improvement in a defined surgical-interface function	Requires input relevance, an encoded transition, off-target stability, interface-level benefit, and matched-control comparison
Tissue-adhesive and translational reviews [5,11]	Wet adhesion mechanisms, interfacial chemistry, multiscale adhesion, temporal or spatial selectivity, tissue environment, and clinical translation	Provides detailed design principles and translational requirements for adhesive materials	Adhesion remains the central behavior and does not fully encompass interfaces requiring low or asymmetric adhesion, temporary separation, lubricity, preserved gliding, or remodeling-permissive integration	Positions adhesion as one function-dependent property within sealing, separation, protection, and integration/reinforcement
Postoperative antiadhesion reviews [4,6]	Mechanisms of adhesion formation, barrier strategies, drug delivery, and clinical evidence	Connects a defined surgical complication with biological mechanisms and preventive strategies	Focuses on postoperative adhesion and does not integrate leakage, washout, tethering, delamination, poor ingrowth, or loss of support	Incorporates postoperative adhesion into a broader failure-mode framework alongside other surgical-interface complications
DNA-hydrogel reviews [14,17,18]	DNA network architecture, backbone rigidity, crosslinking kinetics, functional nucleic-acid motifs, stimulus categories, biosensing, actuation, and release	Establishes sequence-level programmability and molecular control of hydrogel behavior	Surgical mechanics, wet-field handling, procedure-specific failure modes, and function-specific operative endpoints are not the principal organizing axes	Treats DNA as a molecular programming module whose value must be demonstrated within mechanically robust systems and against a defined interface endpoint
Present review	Surgical boundary and failure mode → four core interface functions → material requirements → programmed input–transition–output behavior → comparator and translational evaluation	Provides an integrated reverse-design framework spanning multiple surgical specialties and hydrogel classes	Not applicable	Connects operative complications directly to molecular design priorities, function-specific evidence, and translational claims

The limitations summarized here refer specifically to the purpose of developing a unified, cross-specialty framework for surgical-interface design and do not diminish the contributions of the cited reviews within their intended scopes. The categories are complementary rather than mutually exclusive.

**Table 2:** Function-specific design priorities and evaluation endpoints for surgical-interface hydrogels.

Interface Function and Primary Objective	Design Priorities	Principal Failure Modes	Minimum Evaluation Endpoints
Sealing: Maintain containment of gas, biological fluids, or luminal contents until native repair restores integrity [23,24,25,29,30,31]	Wet-tissue conformity; interfacial adhesion; cohesive durability; pressure/washout resistance; controlled swelling; repair-matched degradation	Persistent leakage; dehiscence; delamination; cohesive rupture; washout; excessive swelling; premature loss of integrity	Leak or burst pressure; leakage rate/volume; adhesion or peel strength; cyclic sealing stability; swelling/volumetric expansion; degradation with residual strength
Separation: Temporarily isolate injured surfaces to reduce unwanted tissue bridging [6,20,32,33,34]	Conformal coverage; selective target retention; low non-target adhesion; antifouling or anti-cell-attachment behavior; dimensional stability; time-limited persistence	Barrier displacement; incomplete coverage; adhesion formation; fibrotic bridging; non-target adhesion; excessive persistence; premature degradation	Surface coverage/retention; adhesion incidence and severity; histological bridging/fibrosis; protein-fouling or cell-adhesion assays; swelling/degradation profile; ease of re-entry
Protection: Preserve gliding, mobility, structural integrity, or neurological function of vulnerable tissues [21,22,35,36,37,38]	Nonconstrictive coverage; lubricity; low friction; mechanical compliance; positional stability; tolerance of repetitive deformation	Restricted gliding; constriction; friction-related injury; scar tethering; pain; neurological or motor dysfunction	Friction coefficient; gliding resistance; tendon excursion or range of motion; electrophysiology or motor-function testing; perineural/peritendinous adhesion scoring; cyclic mechanical testing
Integration/reinforcement: Provide fixation and load transfer while supporting mechanical or biological incorporation [2,26,28,39,40,41,42,43]	Interfacial fixation; cohesive durability; mechanical compatibility; fatigue resistance; controlled persistence/degradation; tissue-ingrowth pathways; favorable host response	Displacement; delamination; seroma; shrinkage; poor tissue incorporation; chronic inflammation; fibrotic encapsulation; loss of support	Fixation or interfacial strength; tensile or burst strength; fatigue resistance; implant displacement/dimensional change; tissue ingrowth/vascularization; capsule thickness/collagen organization

Material requirements and evaluation endpoints are function dependent and may overlap when multiple interface objectives coexist at the same operative site. Testing should be performed under relevant wet, biochemical, and mechanical conditions and, where possible, at multiple time points to capture changes during healing. Hemostatic activity should be mapped to the dominant interface function it supports, most commonly sealing, protection, or reinforcement.

**Table 3:** Operational criteria for clinically meaningful programmability in the surgical-interface context.

Criterion	Requirement	Why It Matters for Surgical Interfaces
Input relevance	The input should be present, enriched, or deliverable at the intended operative site.	A trigger that is absent, nonspecific, or inaccessible cannot reliably control an interface.
Encoded transition	The timing, threshold, spatial localization, sequence, or logic of the material response should be intentionally designed.	Programmability requires more than passive stimulus sensitivity.
Baseline and off-target stability	The material should remain stable under baseline and non-target conditions.	Premature activation may cause leakage, adhesion, loss of support, toxicity, or non-target release.
Interface-level output	The programmed transition should improve a measurable function-specific endpoint.	Relevant endpoints include sealing durability, reduced tissue bridging, preserved gliding, tissue ingrowth, or mechanical reinforcement.
Matched-control comparison	The programmed system should be compared with a nonresponsive or nonprogrammed material with similar baseline properties.	This determines whether the encoded program adds value beyond the underlying hydrogel composition.

These criteria are intended to evaluate claims of programmability, not to rank hydrogel chemistries or stimulus types. The relevant comparator depends on the intended interface function and may include a nonresponsive hydrogel, a scrambled-sequence DNA control, a non-cleavable linker, a trigger-insensitive formulation, or a material with the same payload but without programmed release. Note: The operational criteria summarized here were synthesized from prior frameworks on programmable, stimuli-responsive, and spatiotemporally controlled hydrogels and surgical tissue interfaces [10,11,45,59,60,61,62].

**Table 4:** Compact cross-surgical application map for programmable hydrogel interfaces.

Surgical Context	Dominant Interface Function and Failure Risk	Programmable Design Opportunity	Critical Evaluation Focus
Air, fluid, and luminal sealing interfaces: lung surface, dura, cornea, gastrointestinal anastomosis or perforation	Sealing; leakage, washout, delamination, swelling-related compression, impaired repair, contamination	Organ-matched mechanics; low-swelling adhesion; repair-matched degradation; antimicrobial or anti-inflammatory release; leakage or rebleeding sensing	Compare with matched nonprogrammed analogue and established sealant/patch benchmark; test leak resistance, cyclic stability, washout, swelling safety, repair integrity, and wet-field usability [23,24,25,29,31,42,51,52,53,54,55,56,58,116]
Adhesion-prone surfaces: peritoneal, pericardial, visceral, intrauterine, and implant-adjacent planes	Separation; adhesion formation, fibrotic bridging, non-target adhesion, barrier migration, difficult re-entry	Sprayable or conformal barriers; Janus or asymmetric adhesion/antiadhesion; antifouling surfaces; inflammation or oxidative-stress modulation; time-limited degradation	Test target retention, directional antiadhesion, adhesion severity, tissue bridging, degradation timing, and ease of re-entry against relevant antiadhesion benchmarks [6,20,32,33,34,63,64,65,66]
Motion-preserving vulnerable interfaces: repaired tendons, peripheral nerves, tendon sheaths, nerve tunnels	Protection; restricted gliding, scar tethering, constriction, pain, neurological or motor dysfunction	Lubricating or low-friction surfaces; nonconstrictive wraps; spatially selective retention; anti-inflammatory or antifibrotic release; motion-tolerant degradation	Evaluate gliding resistance, range of motion, electrophysiology or motor function, peritendinous/perineural adhesion, constriction risk, and cyclic deformation [21,22,35,36,37,38,75,76]
Reinforcement or integration interfaces: abdominal wall, mesh–tissue contact, soft-tissue defects, regenerative matrices	Integration/reinforcement; displacement, delamination, seroma, poor tissue ingrowth, fibrosis, loss of support	Controlled fixation; load-sharing mechanics; fatigue resistance; tissue-ingrowth pathways; immunomodulation; degradation matched to remodeling	Test fixation, load transfer, fatigue resistance, tissue ingrowth, vascularization, host response, dimensional stability, and residual mechanical support [2,26,39,40,41,42,43,84,85,86,101,102,103,104,105,106,107]
Infected or impaired-repair operative beds: contaminated wounds, diabetic or ischemic wounds, irregular surgical beds	Protection, repair, or integration support; infection, biofilm, excess inflammation, impaired angiogenesis, poor matrix deposition	Triggered antimicrobial release; ROS or protease-responsive degradation; staged anti-inflammatory, angiogenic, or regenerative outputs; conformal retention	Evaluate retention in irregular beds, bacterial burden or biofilm, inflammatory profile, angiogenesis, matrix deposition, compatibility with drainage/debridement, and systemic safety [80,81,82,83,108,109]
Selected tumor-resection or recurrence-prone cavities	Protection and local disease-control support; residual disease, local recurrence, immune evasion, nonspecific activation	Local depot retention; immunomodulatory, phototherapeutic, or checkpoint-related release; recognition-based surveillance; DNA-based sensing modules	Evaluate local retention, residual disease control, signal specificity, off-target activation, systemic toxicity, compatibility with adjuvant therapy, and feasibility of reoperation [110,111,112,113,114,115]

Categories are not mutually exclusive. A single operative site may require multiple interface objectives, such as sealing with separation, protection with repair, or reinforcement with local biological modulation. This table maps operative contexts to dominant interface functions and programmable opportunities rather than ranking hydrogel chemistries or replacing procedure-specific benchmarks. Biological modulation is treated as an adjunct program layered onto a dominant interface function.

**Table 5:** DNA-based molecular modules mapped to surgical-interface functions and translational constraints.

DNA Module	Encoded Role or Output	Surgical-Interface Relevance	Essential Control and Key Limitation
Sequence-defined networks and DNA crosslinking modules [13,14,16,17,18,117,118,119,120]	Designed hybridization, branched motifs, DNA nanostars, rolling-circle-amplified strands, or strand-displacement motifs can encode self-assembly, gelation, stiffness change, gel–sol transition, degradation, or on-demand release.	Prototype molecular programmability; potential temporal control of release, degradation, or remodeling when combined with mechanically stronger surgical hydrogels.	Controls: noncomplementary sequence, non-exchangeable crosslinker, scrambled sequence, or nonresponsive network with similar baseline mechanics. Limitations: weak mechanics, nuclease susceptibility, trigger delivery, washout, cost, scalability, sterilization, and wet-field handling.
Aptamer-, DNAzyme-, or nuclease-responsive recognition modules [15,17,18,19,121]	Molecular targets, ions, nucleases, pathogens, inflammatory mediators, tumor markers, or disease-associated nucleic acids can trigger binding, capture, release, cleavage, degradation, or signal generation.	Local sensing, targeted release, infection or recurrence surveillance, and triggered degradation or release in biologically active operative beds.	Controls: scrambled aptamer, affinity-mutated aptamer, catalytically inactive DNAzyme, nuclease-resistant linker, noncleavable sequence, or target-absent condition. Limitations: specificity in blood/exudate, target heterogeneity, uncontrolled degradation, degradation-product safety, and off-target activation.
CRISPR-responsive DNA hydrogel modules [15,122]	Guide-defined target recognition coupled to Cas-mediated cleavage or activation can produce target-dependent network degradation, cargo release, or diagnostic signaling.	Pathogen detection, residual tumor or recurrence sensing, and logic-like release in infected or oncologic operative beds.	Controls: no-target condition, scrambled guide RNA, catalytically inactive enzyme, nonresponsive network, or irrelevant nucleic-acid target. Limitations: system complexity, enzyme stability, immune concerns, regulatory complexity, cost, and surgical usability.
CpG or immunostimulatory DNA motifs [110,111,112,113,114,115,123]	Toll-like receptor-recognized CpG motifs or immune-active nucleic-acid sequences can produce local immune activation, adjuvant-like activity, or immune-cell recruitment.	Local immunomodulation in tumor-resection cavities, infected wounds, or recurrence-prone beds when biological control supports protection or local disease control.	Controls: non-CpG sequence, immunologically inert sequence, payload-only control, or hydrogel-only control. Limitations: dose control, systemic immune activation, inflammation-related toxicity, patient variability, and interaction with adjuvant therapy.
DNA-functionalized hybrid or composite hydrogels [13,17,18,103,117]	DNA modules incorporated into PEG, gelatin, hyaluronic acid, alginate, chitosan, or other stronger matrices can add recognition, sensing, release, degradation, or remodeling to adhesive, tough, injectable, or degradable platforms.	Most realistic near-term route for combining molecular programmability with wet-tissue adhesion, handling, and mechanical support.	Controls: hybrid hydrogel without DNA, inactive sequence, matched payload without programmed release, or nonprogrammed material with similar mechanics. Limitations: preserving DNA function after manufacturing, sterilization, storage, delivery, and implantation while maintaining mechanical and adhesive performance.

DNA-based modules are not mutually exclusive and may be combined within hybrid hydrogel or composite systems. DNA sequence programmability should be linked to a defined surgical-interface function and tested against sequence-specific, trigger-insensitive, or nonprogrammed controls with similar baseline material properties. Common translational constraints include nuclease exposure, immune activation, cost and scalability, sterilization compatibility, storage stability, and preservation of DNA function after delivery and implantation.

**Table 6:** Minimum evidence package for translational claims in programmable surgical-interface hydrogels.

Domain	Required Evidence	Comparator/Control	Endpoint	Failure Scenario
Input relevance	Trigger is present, enriched, deliverable, or clinically controllable at the target site; relevant concentration, intensity, timing, and spatial distribution are defined	Baseline and off-target fluids, tissues, or activation conditions	Confirmed trigger exposure matches the intended operative environment	No activation at target site; activation under irrelevant or off-target conditions
Programmed material transition	Response threshold, kinetics, magnitude, reversibility when relevant, and baseline stability are defined	Nonresponsive, trigger-insensitive, noncleavable, scrambled-sequence, or nonprogrammed formulation	Reproducible change in adhesion, degradation, stiffness, permeability, release, surface selectivity, or signal generation	Incomplete activation; premature degradation; excessive swelling; unintended release; loss of asymmetry
Function-specific interface output	Programmed transition improves sealing, separation, protection, or integration/reinforcement	Static or nonprogrammed material with similar baseline mechanics, adhesion, degradation, and payload when applicable	Leak or burst pressure; adhesion reduction; preserved gliding; tissue ingrowth; fixation; load transfer; local biological control	Delamination; washout; recurrent leakage; non-target adhesion; impaired gliding; poor ingrowth; loss of support
Spatial and temporal matching	Output occurs at the intended location and during the relevant healing interval	Uniform material, reversed Janus orientation, delayed or premature activation, or nondegradable control	Correct target-facing behavior; appropriate persistence; repair-matched degradation or remodeling	Misorientation; migration; folding; late persistence; premature clearance; off-target activation
Chronic implant stability, host response, and remodeling	Material and programmed function remain stable through the intended functional lifetime; degradation, mechanical fatigue, host response, and tissue remodeling are evaluated at early, intermediate, and late time points	Matched nonprogrammed material, established implant benchmark, and sham or material-free control when appropriate	Implant position and dimensions; cyclic fatigue; crack growth or delamination; residual mechanical or electrical function; degradation and degradation-product profile; macrophage and foreign-body giant-cell response; capsule thickness and collagen organization; vascularization, tissue ingrowth, native load transfer, and long-term functional outcome	Late delamination or fatigue failure; chronic inflammation; fibrotic encapsulation; seroma; calcification; premature loss of support; persistent nondegraded material; adverse tissue remodeling; late loss of programmed function
Operative usability and failure-safe behavior	Material can be prepared, delivered, activated, visualized, retained, corrected, or removed within the intended workflow	Standard applicator, alternative user, delayed application, wet-field challenge, or minimally invasive deployment condition	Successful deployment; reproducible coverage; retention; acceptable working and curing times	Clogging; runoff; incorrect mixing; incomplete curing; wrong orientation; difficult removal; use-related activation error
Manufacturing, sterilization, and storage	Final-use material preserves baseline properties and programmed behavior after manufacturing, sterilization, packaging, storage, and reconstitution	Pre-sterilization, freshly prepared, aged, and batch-to-batch comparisons	Preserved input sensitivity, response threshold, adhesion, mechanics, degradation, release, and biocompatibility	Loss of trigger response; altered release; payload degradation; endotoxin or residual reagent burden; batch variability
Biological safety and regulatory readiness	Safety is evaluated for baseline, activated, partially activated, degraded, and off-target-exposed material states; primary mode of action is defined	Material-only control, payload-only control, degradation products, and activated versus nonactivated material	Local tissue response; systemic exposure; degradation-product profile; inflammatory, immune, coagulation, or infection-related effects	Unexpected inflammation; activation-related toxicity; unsafe degradation products; uncontrolled release; unclear product classification

The evidence required for translation depends on the intended clinical claim and proposed interface function. A research tool may require proof of encoded response, whereas a surgical sealant, barrier, protective wrap, reinforcing scaffold, local drug-delivery system, or diagnostic-response material requires function-specific interface testing, operative usability assessment, biological safety evaluation, and appropriate regulatory positioning. Note: Representative supporting references for the evidence domains include input–transition–output and comparator logic [10,11,45,59,60,61,62]; chronic stability, mechanical fatigue, host response, and tissue remodeling [39,40,41,42,43,52,84,85,86,100,107]; operative usability and procedure-relevant testing [133,136,137,138,139,140,141,142,143]; and manufacturing, sterilization, regulatory, and clinical translation [5,130,144,145,146,147,148,149,150,151,152,153,154].

**Table 7:** Representative clinically available surgical-interface materials compared with investigational programmable hydrogel strategies.

Material Class and Representative Example	Established Strengths	Principal Limitations	Programmable-Hydrogel Opportunity and Minimum Comparative Evidence
Fibrin-based sealants—e.g., TISSEEL; adjunctive hemostasis and sealing	Clinically familiar clot-mimetic mechanism; conformal application to irregular and wet surfaces; rapid intraoperative use; extensive surgical experience	Limited load-bearing and cohesive performance under high pressure or repetitive motion; biologically derived components and associated preparation or storage requirements; predefined degradation and no adaptive response; not a substitute for structural closure	Hemorrhage-responsive reinforcement, washout-resistant adhesion, localized therapeutic release, or repair-matched degradation. Evaluation should include time to hemostasis, blood loss, leak or burst resistance, cyclic stability, handling, safety, and cost against a fibrin-sealant benchmark [5,27,155].
Polyethylene glycol-based synthetic sealants—e.g., DuraSeal Exact; adjunctive watertight dural sealing	Reproducible synthetic composition; rapid in situ polymerization; conformal defect coverage; effective fluid-barrier formation	Swelling and compression remain important concerns in confined anatomical spaces; limited intrinsic biological activity; predefined mechanics and persistence; performance depends on application thickness, access, and curing workflow	Low-swelling, motion-tolerant, leak-responsive, or repair-matched systems. Comparison should include volumetric expansion, local compression risk, leak pressure, cyclic sealing durability, degradation, neural or tissue safety, and applicator usability [5,56,155].
Albumin–glutaraldehyde adhesives—e.g., BioGlue; adjunctive sealing and hemostasis in large-vessel repair	Rapid and relatively strong tissue bonding; robust fixation on vascular tissue; ready-to-use delivery system; established cardiovascular application	High stiffness relative to native tissue; limited remodeling; potential inflammatory or tissue-injury concerns related to persistent material and aldehyde chemistry; difficult removal after misapplication	Aldehyde-free or dynamically crosslinked adhesives with tissue-matched compliance, controlled degradation, and improved remodeling. Evaluation should include hemostasis, burst resistance, vascular compliance, tissue injury, chronic inflammation, residual material, and long-term remodeling [5,27,155].
Solid bioresorbable antiadhesion barriers—e.g., hyaluronic acid/carboxymethylcellulose membrane (Seprafilm) and oxidized regenerated cellulose barrier (Interceed)	Clinically established physical separation of injured surfaces; localized placement; absorbable formulations; no requirement for active drug release	Product-dependent handling, folding, positioning, and coverage limitations; difficult deployment on irregular or minimally accessible surfaces; migration or incomplete coverage; performance may be sensitive to blood, moisture, and incomplete hemostasis; predefined barrier lifetime and surface behavior	Sprayable or in situ-forming barriers with target-facing retention, non-target-facing antiadhesion, improved conformity, and programmed degradation. Comparison should assess deployment time, coverage, positional stability, blood tolerance, adhesion incidence and severity, tissue bridging, degradation, and ease of re-entry [4,6,156].
Liquid antiadhesion barriers—e.g., 4% icodextrin solution (Adept)	Simple instillation; broad peritoneal distribution; compatibility with laparoscopic application; no need to position a solid sheet	Limited spatial localization and target retention; distribution and residence depend on fluid movement and anatomy; fixed, non-directional barrier action; clinical benefit may vary with procedure and endpoint; fluid-related precautions	Shear-thinning or in situ-gelling systems that combine broad coverage with localized retention and controlled clearance. Comparative studies should assess distribution, residence, fluid balance, adhesion outcomes, operative workflow, and safety [4,6,156].
Programmable hydrogel platforms—predominantly investigational	Potential temporal, spatial, threshold-dependent, or sequential regulation of adhesion, mechanics, degradation, surface behavior, sensing, and release; conformal or minimally invasive delivery; capacity to combine multiple interface functions	Limited clinical evidence; greater manufacturing, sterilization, storage, regulatory, and cost complexity; risk of off-target or premature activation; trigger-access and reliability constraints; possible dependence on specialized applicators or activation devices	Added complexity is justified only when a programmed transition improves a procedure-specific interface endpoint. Each platform should be tested against both a matched nonprogrammed analogue and the best available clinical benchmark, including operative usability, safety, manufacturing reproducibility, shelf life, cost, and clinically meaningful functional outcomes [10,11,45].

Representative products are included as illustrative clinical benchmarks rather than as an exhaustive market survey or an endorsement of a specific product. Availability, regulatory classification, labeling, and approved indications vary by jurisdiction. Programmable hydrogel platforms remain predominantly investigational and should not be considered superior unless they demonstrate a clinically meaningful advantage over both a compositionally matched nonprogrammed control and an established procedure-specific benchmark.

## Data Availability

No new data were created or analyzed in this study. Data sharing is not applicable to this article.

## Abbreviations

The following abbreviations are used in this manuscript:

Cas12a	CRISPR-associated protein 12a
CpG	cytosine-phosphate-guanine
CRISPR	clustered regularly interspaced short palindromic repeats
DNA	deoxyribonucleic acid
ECM	extracellular matrix
GC	guanine-cytosine
MMP	matrix metalloproteinase
ROS	reactive oxygen species
